# Altered Brain‐Behavior Association During Resting State is a Potential Psychosis Risk Marker

**DOI:** 10.1002/advs.202405700

**Published:** 2025-04-02

**Authors:** Leonardo Fazio, Giuseppe Stolfa, Roberta Passiatore, Angelantonio Tavella, Giuseppe Blasi, Madalina O. Buciuman, Aaron L. Goldman, Shalaila S. Haas, Lana Kambeitz‐Ilankovic, Nikolaos Koutsouleris, Monica Nicoli, Teresa Popolizio, Antonio Rampino, Anne Ruef, Fabio Sambataro, Pierluigi Selvaggi, William Ulrich, Daniel R. Weinberger, Apulian Network on Risk for Psychosis, PRONIA Consortium, Alessandro Bertolino, Linda A. Antonucci, Giulio Pergola

**Affiliations:** ^1^ Department of Medicine and Surgery LUM University Casamassima Bari 70010 Italy; ^2^ Department of Translational Biomedicine and Neuroscience University of Bari Aldo Moro Bari 70124 Italy; ^3^ Lieber Institute for Brain Development Johns Hopkins Medical Campus Baltimore MD 21205 USA; ^4^ Psychiatry Unit University Hospital Bari 70124 Italy; ^5^ Department of Psychiatry and Psychotherapy Ludwig‐Maximilian‐University 80336 Munich Germany; ^6^ Department of Psychiatry and Psychotherapy Faculty of Medicine and University Hospital University of Cologne 50931 Cologne Germany; ^7^ Max‐Planck Institute of Psychiatry 80804 Munich Germany; ^8^ Institute of Psychiatry, Psychology and Neuroscience King's College London London SE5 8AF UK; ^9^ Neuroradiology Unit Scientific Institute for Research, Hospitalization and Health Care Casa Sollievo della Sofferenza San Giovanni Rotondo Foggia 71013 Italy; ^10^ Section of Psychiatry Department of Neuroscience University of Padova Padua 35121 Italy; ^11^ Department of Neuroimaging Institute of Psychiatry, Psychology and Neuroscience King's College London London SE5 8AF UK; ^12^ Department of Neurology and Radiology Johns Hopkins Medical Campus Baltimore MD 21205 USA; ^13^ Department of Psychiatry and Behavioral Sciences Johns Hopkins University School of Medicine Baltimore MD 21205 USA; ^14^ Department of Neuroscience Johns Hopkins University School of Medicine Baltimore MD 21205 USA; ^15^ Department of Genetic Medicine Johns Hopkins University School of Medicine Baltimore MD 21205 USA

**Keywords:** at risk mental states, brain networks, brain‐behavior relationship, chronic psychosis, executive function, neuropsychology

## Abstract

Alterations in cognitive and neuroimaging measures in psychosis may reflect altered brain‐behavior interactions patterns accompanying the symptomatic manifestation of the disease. Using graph connectivity‐based approaches, we tested the brain‐behavior association between cognitive functioning and functional connectivity at different stages of psychosis. We collected resting‐state fMRI of 204 neurotypical controls (NC) in two independent cohorts, 43 patients with chronic psychosis (PSY), and 22 subjects with subthreshold psychotic symptoms (STPS). In NC, we calculated graph connectivity metrics and tested their associations with neuropsychological scores. Replicable associations were tested in PSY and STPS and externally validated in three cohorts of 331, 371, and 232 individuals, respectively. NC showed a positive correlation between the degree centrality of a right prefrontal‐cingulum‐striatal circuit and total errors on Wisconsin Card Sorting Test. Conversely, PSY and STPS showed negative correlations. External replications confirmed both associations while highlighting the heterogeneity of STPS. Group differences in either centrality or cognition alone were not equally replicable. In four independent cohorts totaling 1,203 participants, we identified a replicable alteration of the brain‐behavior association in different stages of psychosis. These results highlight the high replicability of multimodal markers and suggest the opportunity for longitudinal investigations that may test this marker for early risk identification.

## Graphical abstract

The study detects a potential multimodal biomarker that can be promising for identifying early markers of psychosis. It shows a consistent brain‐behavior association between a circuit of interconnected regions and executive function in neurotypical controls and individuals at various stages of psychosis. These findings are supported by data from four independent cohorts, totaling 1,203 participants.

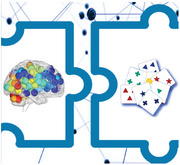

## 1 Introduction

Each human brain has unique structure, activity, and connectivity patterns. These dimensions are so distinctive that they create a neural “fingerprint” capable of differentiating one individual from another.^[^ ^1^ ^]^ This remarkable variability in brain structural, activity, and connectivity profiles not only characterizes individuals, but also underlies differences in cognition and behavior, in terms of intelligence, personality, or emotional functioning.^[^ ^1^, ^2^ ^]^ While brain and cognitive/behavioral profiles have often been associated with each other, the relationship between them, i.e., patterns emerging at the level of brain/cognition correlations rather than in one or the other modality, has been reported more rarely in the context of understanding inter‐individual differences. Besides illuminating the functional correlates of brain activity at the group level, combined brain/cognitive investigations are relevant to generating individual patterns with potential marker value in clinical applications.

In nonclinical samples, individual profiles of functional connectivity based on fMRI data predict scores on cognitive tasks that measure working memory,^[^ ^3^ ^]^ attention,^[^ ^4^ ^]^ executive function,^[^ ^5^ ^]^ fluency,^[^ ^5^, ^6^ ^]^ or intelligence,^[^ ^7^ ^]^ as well as personality,^[^ ^8^ ^]^ and decision impulsivity.^[^ ^9^ ^]^ In clinical samples, overt performance deficits usually dominate brain‐behavior relationships, entangling the interpretations of functional connectivity deficits. Indeed, chronic patients with psychosis report not only impaired cognition, particularly in executive function,^[^ ^10^ ^]^ attention,^[^ ^10^, ^11^ ^]^ speed of information processing,^[^ ^11^, ^12^ ^]^ and working memory,^[^ ^12^, ^13^ ^]^ but also altered functional brain connectivity.^[^ ^14^ ^]^ An intriguing perspective is that brain alterations in the absence of deficits may predate overt cognitive impairments. For example, the study of intermediate phenotypes of schizophrenia has revealed heritable brain alterations in individuals at genetic risk for schizophrenia who are otherwise healthy.^[^ ^15^ ^]^

In this framework, brain connectivity is promising for biomarker identification, as several studies have shown in relation to cognitive functions such as working memory, attention, or processing speed in psychotic spectrum disorders.^[^ ^3^, ^14^, ^16^ ^]^ Previous reports described alterations in functional connectivity estimated via voxelwise coupling in association with cognition before the onset of psychotic symptoms.^[^ ^17^ ^]^ Independent component analysis revealed early alterations in functional network connectivity associated with cognition.^[^ ^18^ ^]^ Such patterns might, therefore, serve to detect at risk individuals before their signs and symptoms meet diagnostic criteria.

As interindividual variability in cognition reflects in part the variability in brain connectivity,^[^ ^19^ ^]^ it has been reported that even resting state connectivity patterns predict the performance of cognitive functions such as attention,^[^ ^16b^ ^]^ processing speed,^[^ ^16a,b^ ^]^ working memory^[^ ^16a^ ^]^ or episodic memory,^[^ ^16c^ ^]^ as measured by neuropsychological tasks performed outside the scanner. The link between resting state and cognitive performance suggests that intrinsic connectivity patterns, unrelated to task performance, may be associated with cognitive, clinical, or genetic variables.^[^ ^18^, ^20^ ^]^

In this study, we set out to identify replicable brain‐behavior associations using resting state network features. Most studies focused on seed‐based or a priori‐selected network connectivity analyses.^[^ ^14^, ^16^, ^17^, ^21^ ^]^ These approaches only capture a portion of the system‐level function.^[^ ^22^ ^]^ Instead, approaches based on graph theory consider the whole brain as a network composed of nodes and edges, that is, brain regions and the relationships between them.^[^ ^22^, ^23^ ^]^ These measures capture network properties and interactions at the micro‐, meso‐, and macro‐scale level, accounting for both global signal and relationships among brain regions.^[^ ^23^, ^24^ ^]^ Unlike independent component analysis, graph theory preserves region‐specific attributes.

Several studies have reported alterations in graph properties in patients with psychosis,^[^ ^25^ ^]^ in subjects with subthreshold psychotic symptoms,^[^ ^25^, ^26^ ^]^ and in first‐degree relatives of patients with psychosis, such as unaffected siblings.^[^ ^27^ ^]^ In more detail, analyses based on graph theory allow the investigation of specific features of the topological organization of brain connectivity, such as degree centrality, i.e., the number of connections a node makes with other nodes.^[^ ^28^ ^]^ Using this analogy, the brain can be viewed as a complex, hierarchical topological network with interactions at the micro‐, meso‐, and macro‐scale levels. Measures derived from graph theory assess the efficiency and integration of communication within this topological network, by identifying hubs and interconnected brain structures.^[^ ^29^ ^]^ This serves to investigate disorders associated with alterations related to how information is integrated and flows within the brain network.^[^ ^30^ ^]^

Previous studies have revealed that brain measures of centrality vary between states associated with psychosis: they differ between individuals with chronic psychosis,^[^ ^31^ ^]^ early episodes,^[^ ^32^ ^]^ subthreshold psychotic symptoms,^[^ ^31^, ^33^ ^]^ and unaffected siblings of patients.^[^ ^34^ ^]^ Notably, alterations in measures of brain centrality have been associated with impairments in cognitive functions, such as working memory or executive functions, in both clinical^[^ ^35^ ^]^ and nonclinical populations.^[^ ^36^ ^]^ This evidence suggests functional alterations in the way the brain processes information, which could predict clinical symptomatology.

**Figure 1:**
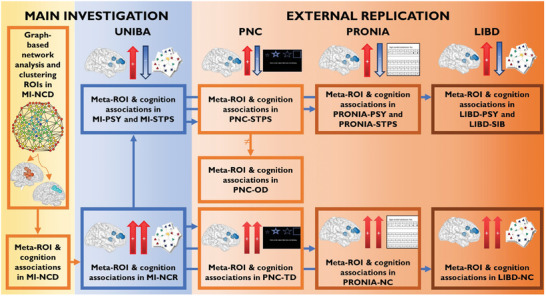
Outline of the study. The main phases of the study are shown in the figure. For each cohort, the arrows between the brain and the cognitive test indicate the directionality of the association (*pFDR <* 0.05) between the degree centrality of right prefrontal‐cingulum‐striatal brain regions and cognitive functioning. Arrows pointing in the same direction represent a positive brain‐behavior association, whereas arrows pointing in opposite directions represent a negative brain‐behavior relationship. Abbreviations: MI: Main investigation of the study using UNIBA dataset (*n = *269); MI‐NCD: Neurotypical controls in discovery cohort of the main investigation (*n = *117); MI‐NCR: Neurotypical controls in within‐site replication cohort of the main investigation (*n = *87); MI‐PSY: Patients with psychosis of the main investigation (*n = *43); MI‐STPS: Individuals with subthreshold psychotic symptoms of the main investigation (*n = *22); PNC: Philadelphia Neurodevelopmental Cohort (*n = *331); PNC‐TD: Individuals with typical development in PNC dataset (*n = *57); PNC‐STPS: Individuals with a developmental trajectory toward psychotic disorders in PNC dataset (*n = *45); PNC‐OD: Individuals with a developmental trajectory toward other psychiatric disorders in PNC dataset (*n = *229); PRONIA: Personalised Prognostic Tools for Early Psychosis Management dataset (*n = *371); PRONIA‐NC: Neurotypical controls in PRONIA dataset (*n = *202); PRONIA‐PSY: Patients with psychosis in PRONIA dataset (*n = *80); PRONIA‐STPS: Individuals with subthreshold psychotic symptoms in PRONIA dataset (*n = *89); LIBD: Lieber Institute for Brain Development (*n = *232); LIBD‐NC: Neurotypical controls in LIBD dataset (*n = *149); LIBD‐PSY: Patients with psychosis in LIBD dataset (*n = *54); LIBD‐SIB: Unaffected siblings of patients with schizophrenia in LIBD dataset (*n = *29).

Here, we employed measures of centrality and cognitive scores to investigate brain‐behavior relationships. This approach considers brain‐behavior relationships as the primary object of investigation, under the hypothesis that the relationships themselves, rather than their individual brain and/or behavioral components,^[^ ^25^, ^37^ ^]^ may represent potential illness markers. After identifying robust patterns of brain‐behavior associations between centrality measures and cognitive scores, we assessed the replicability of the candidate associations in an independent sample of neurotypical controls (NC), as well as in three external validation samples. We used the Philadelphia Neurodevelopmental Cohort (PNC), the multi‐site European PRONIA study (Personalised Prognostic Tools Early Psychosis Management; www.pronia.eu), and the NIH SIB‐Study dataset available at the Lieber Institute for Brain Development (LIBD). As secondary objectives, the replicability of these correlations was investigated (i) in clinical populations, to test whether neurotypical patterns of brain‐behavior association between centrality measures and cognitive scores were altered both in patients (PSY) with a chronic diagnosis of schizophrenia or bipolar disorder with psychosis, and (ii) in subjects carrying risk for psychosis. Specifically, we selected a population of subjects carrying clinical risk, who manifested subthreshold psychotic symptoms (STPS), and a population of subjects carrying familial risk, i.e., unaffected siblings of patients with psychosis (SIB). The rationale for this approach was that an effect present only in the clinical population, without a physiological correlate in controls, could be driven by confounding variables related to clinical status, such as pharmacological treatment. Instead, a replicable brain‐behavior relationship in controls was expected to reflect brain features supporting cognitive performance. Similar to what was done in NC, we replicated in external validation the results of clinical samples, using PNC, PRONIA, and LIBD datasets. We hypothesized that the detected brain‐behavior relationships could be sensible and replicable features for the identification of subjects with subthreshold psychotic symptoms and the unaffected siblings of patients with psychosis. The study design is depicted in **Figure** **1**.

## 2 Results

### 2.1 Identification of Meta‐ROIs

**Figure 2:**
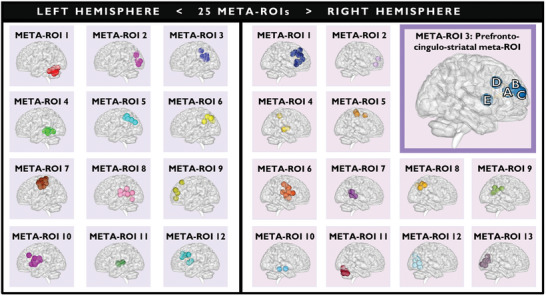
The 25 meta‐ROIs obtained through cluster analysis in MI‐NCD. Hierarchical Ward clustering analysis was performed using the coordinates of 144 ROIs from the Dosenbach atlas (x, y, z based on the MNI atlas), along with the mean values of ROI‐betweenness centrality and ROI‐degree centrality, calculated in MI‐NCD cohort (*n = *117). More details are reported for meta‐ROI 3 of the right hemisphere, i.e., the prefrontal‐cingulum‐striatal meta‐ROI. Capital letters refer to brain regions included in the prefrontal‐cingulum‐striatal meta‐ROI: A. Anterior cingulate cortex; B. Superior frontal gyrus; C. Middle frontal gyrus; D. Cingulate gyrus; E. Caudate. Meta‐ROIs displayed using BrainNet Viewer. Abbreviation: MI‐NCD: Neurotypical controls in discovery cohort of the main investigation.

The analysis of graph‐based functional connectivity measures was first carried out in non‐clinical samples (both NC cohorts of the main investigation) to identify normative brain‐behavior patterns. We focused our investigation on 144 distinct regions of interest (ROIs), identified by the Dosenbach atlas,^[^ ^38^ ^]^ and for each ROI we calculated two global graph‐based centrality metrics, namely the degree centrality and betweenness centrality. Adjacent ROIs displayed similar mean centrality values throughout the brain, creating spatial clusters of centralities. Therefore, we clustered the ROIs based on their functional similarity and spatial proximity, resulting in the generation of “meta‐ROIs”. This procedure was performed using hierarchical Ward clustering^[^ ^39^ ^]^ analysis, which utilized the coordinates of the ROIs (x, y, z according to the MNI atlas) and the mean values of the ROI‐betweenness centrality and ROI‐degree centrality previously calculated in NC subjects. The entire procedure is detailed below in the Experimental Section. We clustered ROIs separately for the left and right hemispheres and cut the dendrogram at the lowest level.^[^ ^40^ ^]^ We obtained 25 meta‐ROIs (**Figure** **2**), 12 in the left and 13 in the right hemisphere.

### 2.2 Association between Meta‐ROIs Centrality Measures and Cognitive Functioning: Main Investigation

#### 2.2.1 Identification of Neurotypical Patterns of Brain‐Behavior Association

**Figure 3:**
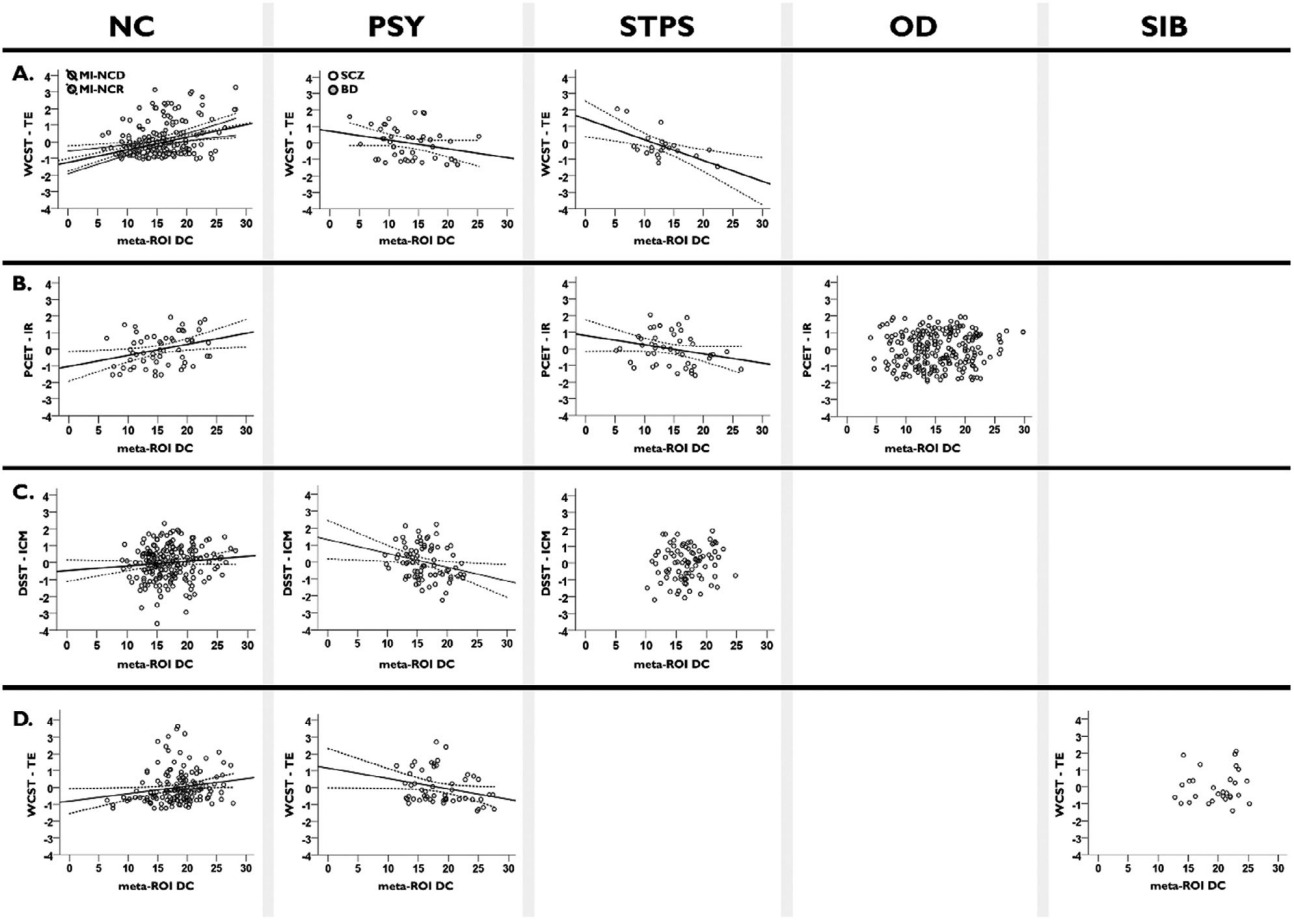
Association between the degree centrality of the prefrontal‐cingulum‐striatal meta‐ROI and the executive performance. A) Association between the meta‐ROI degree centrality and the standardized residuals WCST‐TE in main investigation (*pFDR <* 0.05). B) Association between the meta‐ROI degree centrality and the standardized residuals PCET‐IR in PNC dataset (*P < *0.05). Individuals with typical development (PNC‐TD) are plotted in the first box on the left, in the NC column. Individuals with a developmental trajectory toward psychotic disorders (PNC‐STPS) are plotted in the third box on the left, in the STPS column. C) Association between the meta‐ROI degree centrality and the standardized residuals DSST‐ICM in PRONIA dataset (*P < *0.05). D) Association between the meta‐ROI degree centrality and the standardized residuals of WCST‐TE in LIBD dataset (*P < *0.05). In graphs are plotted the 95% confidence intervals. Abbreviations: NC: Neurotypical controls; PSY: Patients with psychosis; STPS: Individuals with subthreshold psychotic symptoms; OD: Individuals with a developmental trajectory toward psychiatric disorders other than psychosis in PNC dataset (*n = *229); SIB: Unaffected siblings of patients with schizophrenia in LIBD dataset (*n = 29*); MI‐NCD: Neurotypical controls in discovery cohort of the main investigation (*n = *117); MI‐NCR: Neurotypical controls in within‐site replication cohort of the main investigation (*n = *87); SCZ: Patients with schizophrenia (*n = *24); BD: Patients with bipolar disorder (*n = *19); PNC: Philadelphia Neurodevelopmental Cohort (*n = *331); PNC‐TD: Individuals with typical development in PNC dataset (*n = *57); PNC‐STPS: Individuals with a developmental trajectory toward psychotic disorders in PNC dataset (*n = *45); PRONIA: Personalised Prognostic Tools for Early Psychosis Management dataset (*n = *371); LIBD: Lieber Institute for Brain Development (*n = *232); DC: Degree centrality; WCST‐TE: Number of total errors at Wisconsin Card Sorting Test; PCET‐IR: Proportion of incorrect responses at Penn Conditional Exclusion Test; DSST‐ICM: Inverted T‐score of correct symbol‐number matching at Digit Symbol Substitution Test.

Amongst the identified meta‐ROIs, the NC discovery cohort showed a significant positive correlation between the degree centrality of a right prefrontal‐cingulum‐striatal meta‐ROI (Figure 2) and the total error number at the Wisconsin Card Sorting Test (WCST‐TE; *r* = 0.33; *pFDR* = 0.038). The prefrontal‐cingulum‐striatal meta‐ROI included five Dosenbach ROIs, specifically: anterior cingulate cortex (*x,y,z* = 9,39,20); superior frontal gyrus (*x,y,z* = 29,57,18); middle frontal gyrus (*x,y,z* = 27,49,26); cingulate gyrus (*x,y,z* = 9,20,34); caudate nucleus (*x,y,z* = 14,6,7). The same brain‐behavior association was significant in the NC replication cohort (*r* = 0.27; *P* = 0.007). Correlation plots are shown in **Figure** **3**. Also, the slope comparison between correlations found in both NC cohorts (*Z* = 0.49; *P* = 0.625) showed no significant difference. No replicable associations were found between the betweenness centrality of the meta‐ROIs and neuropsychological scores. Sex effects in the association between the degree centrality of the prefrontal‐cingulum‐striatal meta‐ROI and the executive performance are reported in the Section S2.4 (Supporting Information). Validation analysis with the Automatic Anatomical Labeling (AAL) atlas^[^ ^41^ ^]^ identified a meta‐ROI including areas in the right cingulate and prefrontal cortices, which was the most comparable with the prefrontal‐cingulum‐striatal meta‐ROI described above. This AAL meta‐ROI confirmed the positive correlation between degree centrality and WCST‐TE based on an independent anatomical parcellation (*r* = 0.16; *P* = 0.046; Section S2.1, Supporting Information).

#### 2.2.2 Alteration of Brain‐Behavior Association in Clinical Populations

The replicable brain‐behavior association, previously identified in the NC cohorts, was tested in the clinical cohorts. The association between the degree centrality of the right prefrontal‐cingulum‐striatal meta‐ROI and WCST‐TE scores showed negative correlations in the PSY and STPS cohorts (*r* = ‐0.37; *P* = 0.019 and *r* = ‐0.59; *P* = 0.007, respectively). Figure 3 illustrates this relationship. Fisher's r to z test revealed no significant difference between PSY and STPS correlations (*Z* = 1.07; *P* = 0.287), but large differences between PSY and NC discovery / replication cohorts (*Z* = ‐3.98; *P* = 0.001 and *Z* = ‐3.45; *P* = 0.001, respectively) as well as STPS and NC discovery / replication cohorts (*Z* = ‐4.18; *P* = 0.001 and *Z* = ‐3.81; *P* = 0.001, respectively). Sex effects in the association between the degree centrality of the prefrontal‐cingulum‐striatal meta‐ROI and the executive performance are reported in the Section S2.4 (Supporting Information). Validation analysis with the AAL atlas confirmed the negative correlation between degree centrality and WCST‐TE in both clinical groups (PSY: *r* = ‐0.29; *P* = 0.033 and STPS: *r* = ‐0.41; *P* = 0.043; Section S2.1, Supporting Information).

As a sanity check, we explored associations between centrality measures of all 25 meta‐ROIs and all neuropsychological scores (see Sections S1.8 and S2.5, and Tables S2a–d, Supporting Information) in the clinical cohorts, regardless of whether any relationship was significant in NC cohorts. In the PSY cohort, we found that the degree centrality of a right ventromedial prefrontal meta‐ROI (meta‐ROI 2 in Figure 2) correlated negatively with WCST‐TE scores (*r* = ‐0.59; *pFDR* = 0.007) and positively with the total Wechsler Memory Scale score *(r* = 0.53; *pFDR* = 0.029). These effects were not significant in the STPS cohort *(r =* ‐0.17*; pFDR =* 0.995 and *r =* 0.39; *pFDR =* 0.995). We successfully replicated the latter correlation in the LIBD clinical cohort, as described below, suggesting that this brain‐behavior relationship was replicable in chronic patients but did not characterize either NC or STPS cohorts.

#### 2.2.3 Investigations on Centrality Measure and Cognitive Performance

The ANOVA exploring the difference between NC replication cohort, PSY, and individuals with STPS in the degree centrality of the prefrontal‐cingulum‐striatal meta‐ROI returned no significant difference between samples (*P* > 0.05). However, the three cohorts differed regarding WCST‐TE scores (*F*(2,149) = 26; *P* = 0.001). Bonferroni's post‐hoc results showed poorer executive performance in PSY compared to NC replication cohort (*t*(128) = ‐7.20; *P* = 0.001) and individuals with STPS (*t*(63) = ‐2.42; *P* = 0.018). No difference between NC replication cohort and subjects with STPS was significant. To investigate the potential specificity of our brain‐behavior potential marker compared with single brain‐ or behavior‐based measures, we analyzed group‐level differences in whole‐brain centrality measures for all 25 meta‐ROIs, as well as group‐level differences in neuropsychological indices. All results are detailed in the Sections S1.8 and S2.5, and Table S3 (Supporting Information). As concerns meta‐ROIs, we found primarily between‐group differences in degree centrality, such that degree centrality was lower in clinical samples compared with neurotypical samples. However, only group differences in a right ventromedial prefrontal meta‐ROI (meta‐ROI 2 in Figure 2) were externally replicated in the PRONIA cohort, as described below. Analyses on neuropsychological scores showed group‐level differences across all domains, with poorer performance in PSY compared to NC for all scores, and poorer performance of STPS compared with NC on the Wechsler Memory Scale, N‐Back, and Trail Making Test scores. We partially replicated between‐group differences at the Wechsler Memory Scale (*F(2,139)* = 31.07; *pFDR =* 0.001) in the LIBD cohort, as well as between‐group differences at the N‐Back test (*F(2,368)* = 14.91; *pFDR* = 0.001) in the PRONIA cohort.

### 2.3 Association between Meta‐ROIs Centrality Measures and Cognitive Functioning: External Replication

In the external replication phase, we found, in the PNC cohort, a positive correlation (*r* = 0.31; *P* = 0.011) between the proportion of incorrect responses (PCET‐IR) responses at Penn Conditional Exclusion Test (PCET) and the degree centrality of the prefrontal‐cingulum‐striatal meta‐ROI in a group of individuals with typical development (PNC‐TD). Conversely, the same analysis in a group of individuals with a developmental trajectory toward psychotic disorders (PNC‐STPS) indicated a negative correlation (*r* = ‐0.26; *P* = 0.044; Figure 3). The association in a group of individuals with a developmental trajectory toward other psychiatric disorders (PNC‐OD) was not significant despite the larger size of this group (*r* = 0.07; *P* = 0.153; Figure 3). No difference was found between groups in terms of either degree centrality or performance at PCET (*P* > 0.05).

Similarly, in the PRONIA cohort, we found a positive correlation (*r* = 0.12; *P* = 0.047) between the inverse number of correct symbol‐number correspondences (DSST‐ICM) at Digit Symbol Substitution Test and the degree centrality of the prefrontal‐cingulum‐striatal meta‐ROI in the PRONIA‐NC group. Conversely, we found again a negative correlation between DSST‐ICM and degree centrality in the PRONIA‐PSY group (*r* = ‐0.27; *P* = 0.008). The association in the PRONIA‐STPS group was not significant (*r* = 0.14; *P* = 0.096; Figure 3). The two‐sample t‐test exploring the difference in connectivity between PRONIA‐NC and PRONIA‐PSY returned no significant difference in the degree centrality of the prefrontal‐cingulum‐striatal meta‐ROI (*P* > 0.05). However, the PRONIA‐NC cohort revealed a lower DSST‐ICM (*t*(280) = ‐10.48; *P* = 0.001) compared to PRONIA‐PSY in the two‐sample t‐test performed on executive performance. In addition, we found group differences in the degree centrality of a right ventromedial prefrontal meta‐ROI (meta‐ROI 2) (*F(2,368)* = 5.98, *pFDR* = 0.035). Post‐hoc results showed a greater degree centrality in PRONIA‐NC compared with PRONIA‐PSY, and no difference between these groups and PRONIA‐STPS (Table S3, Supporting Information).

Finally, in the LIBD cohort, we found again a positive correlation (*r* = 0.19; *P* = 0.012) between the WCST‐TE and the degree centrality of the prefrontal‐cingulum‐striatal meta‐ROI in the LIBD‐NC group. Conversely, we found again a negative correlation between WCST‐TE and degree centrality in the LIBD‐PSY group (*r* = ‐0.29; *P* = 0.019). The association in the LIBD‐SIB group was not significant (*r* = 0.09; *P* = 0.657; Figure 3). The two‐sample t‐test exploring the difference between LIBD‐NC and LIBD‐PSY in the connectivity of the meta‐ROI returned no significant difference between samples (*P* > 0.05). However, the two‐sample t‐test performed on WCST‐TE showed poorer executive performance in LIBD‐PSY compared to LIBD‐NC (*t*(201) = ‐7.46; *P* = 0.001). Finally, we found a negative correlation between the right ventromedial prefrontal meta‐ROI (meta‐ROI 2) and the Wechsler Memory Scale score (*r* = 0.29; *P* = 0.031). Sex effects in the association between the degree centrality of the prefrontal‐cingulum‐striatal meta‐ROI and the executive performance are reported in the Section S2.4 (Supporting Information).

## 3 Discussion

This study sought to identify a replicable pattern of altered brain–behavior relationships in psychosis. The results showed a replicable brain‐behavior association between degree centrality and a heterogeneous set of tests related to executive function. Between‐group differences in degree centrality or executive function on their own were not equally replicable. The results were also partially replicated in the independent cohorts of PNC, PRONIA, and LIBD, despite the differences between the executive function measures considered.

In NC, lower degree centrality in this meta‐ROI was associated with better cognitive performance, while the opposite trend characterized all three clinical psychosis groups and two of the three subclinical psychosis groups. These relationships were statistically significant for certain cognitive measures associated with psychosis, but not for others equally representative of cognitive deficits in psychosis.

Our data can be interpreted in line with prior work that the connectivity at resting state of regions included in this meta‐ROI stratifies individuals in terms of certain aspects of executive function performance. Indeed, the connectivity of the superior and middle frontal gyrus,^[^ ^42^ ^]^ cingulate gyrus,^[^ ^43^ ^]^ and caudate nucleus,^[^ ^44^ ^]^ has been associated with executive functioning across diverse connectivity methods. These regions play a relevant role in functional brain networks associated with cognitive flexibility, working memory, attention, problem solving, and reasoning, being part of the lateral frontal‐parietal and frontal‐striatal circuits.^[^ ^45^ ^]^

Our findings on NC report better performance associated with a lower degree centrality of the prefrontal‐cingulum‐striatal meta‐ROI during the resting state across five independent samples. Figure 5 shows that the degree centrality of this meta‐ROI is relatively low compared to other meta‐ROIs, during resting state. Thus, in the healthy samples, the connectivity of this meta‐ROI with the rest of the brain involves fewer nodes, or weaker connections with the same number of nodes compared to other meta‐ROIs. This is especially apparent in high‐performing individuals than in the rest of the cohort. To interpret these results, we need to consider that the prefrontal‐cingulum‐striatal meta‐ROI includes “task‐active” regions that increase their activity during task performance and decrease it during rest.^[^ ^46^ ^]^ A possible interpretation is that healthy individuals with higher executive performance effectively disengage task‐active regions during resting state. Low‐performers in the healthy samples show less effective disengagement, thus higher connectivity in task‐active regions during resting state. Accordingly, previous reports show that hypoconnected brain states during resting state are associated with superior Wisconsin Card Sorting Test scores.^[^ ^45^, ^47^ ^]^

Analyses conducted in clinical samples (PSY and individuals with STPS) revealed an opposite pattern. In these groups, higher degree centrality of the prefrontal‐cingulum‐striatal meta‐ROI was associated with better performance at the Wisconsin Card Sorting Test. This reversal was replicable in all samples of chronic patients across the datasets included in our study.

Several studies have reported in PSY, compared with NC, lower global connectivity^[^ ^48^ ^]^ during resting state, as well as lower connectivity of prefrontal regions with the rest of the brain.^[^ ^49^ ^]^ Moreover, a substantial body of evidence described deficits in executive functions in PSY patients.^[^ ^10^, ^50^ ^]^ Taken together, the literature supports the idea that patients with higher connectivity are less impaired and that this higher connectivity is functional to a better cognitive performance. Patients with even lower connectivity are more impaired and may instead make more errors, thus explaining the negative relationship we found. This inverse correlation may be indicative of impaired connectivity in patients, as we also observed in other prefrontal meta‐ROIs, such as the right meta‐ROI 2: here, lower degree centrality was associated with poorer performance on the Wechsler Memory Scale, in chronic patients.

**Figure 4:**
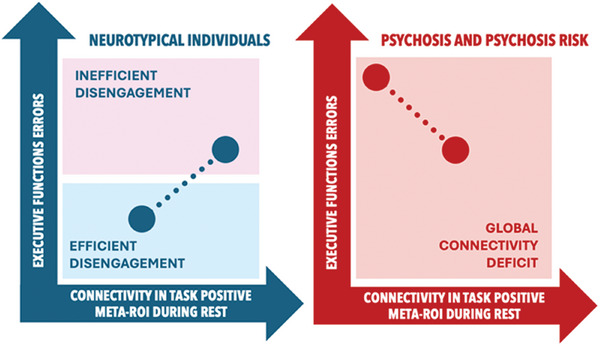
Graphical representation of the interpretative model. In NC, the reduction in connectivity reflected a functional response to the rest condition, indicating an efficient disengagement of task‐dependent regions. In contrast, clinical populations exhibited reduced connectivity, suggesting pathological dysconnectivity of networks, where individuals with less compromised brain networks showed better performance compared to those with global dysconnectivity.

A possible interpretation of these results is that in NC, the reduction in connectivity is a functional response to the rest condition and consists of an efficient disengagement of task‐dependent regions. In contrast, in clinical populations, reduced connectivity may relate to pathological dysconnectivity of networks, so individuals with less compromised brain networks will have more preserved performance than patients with global dysconnectivity. To more comprehensively illustrate this interpretive model, **Figure** **4** presents a schematic graphical representation. The identification of an optimal state of prefrontal‐striatal networks in relation to executive function is reminiscent of the inverted‐U hypothesis of dopaminergic transmission in this brain circuit,^[^ ^51^ ^]^ which was developed to explain task performance.

Notably, individuals with STPS exhibit patterns of brain‐behavior association similar to those of PSY. These findings are in line with previous evidence indicating an alteration of connectivity patterns in individuals with STPS and early illness schizophrenia individuals.^[^ ^52^ ^]^ Indeed, participants with STPS may already experience inefficiency of the prefrontal‐cingulum‐striatal regions essential for task performance.^[^ ^53^ ^]^ This inefficiency is not so pronounced as to impair executive function, which would explain the absence of performance differences compared to the two NC cohorts of the main investigation. The fact that the reversal of the association pattern between degree centrality and performance is detectable across multiple independent cohorts confirms the value of multimodal markers in the context of early identification. We speculate that, like PSY, individuals with STPS also show a reduction of global connectivity during resting state: these changes in connectivity are not severe enough to generate a cognitive deficit but are nevertheless captured when jointly evaluating brain and behavior.

On the other hand, it should be expected that in the more heterogeneous group of at‐risk subjects, not all of them fit this model. This is intrinsic to the definition of at‐risk state, as only a fraction of these patients will transition to chronic psychosis. Such heterogeneity may explain why the brain‐behavior association has not been replicated in PRONIA‐STPS (Section S2.3 and Table S4, Supporting Information).

In summary, our work proposed a rigorous approach to identify a replicable brain‐behavior association that is altered in psychosis and has the potential to qualify as a marker of disease. The brain‐behavior pattern examined in this study was derived from a selection process based on the presence of an association between measures of centrality and cognitive scores in both cohorts of neurotypical controls included in the main investigation, which was then explored in the patient samples, revealing a consistent alteration that may be tested in the future as a marker of disease.

We identified a single neurotypical brain‐behavior pattern. However, this does not preclude the possibility that other brain‐behavior associations were present in both neurotypical controls and clinical samples (Table S2, Supporting Information). Furthermore, the approach proposed offers a parsimonious method for studying brain‐behavior associations that may be relevant to psychosis. Indeed, the measures employed in this study rely on simple and rapid paper‐and‐pencil neuropsychological tests, which are commonly administered in clinical practice, and a relatively basic resting‐state fMRI acquisition sequence. In addition, the replicability of our results on different samples speaks in favor of the robustness of the methodology employed, which thus constitutes a parsimonious and reliable approach.

We hypothesize that future investigations of this candidate marker may reveal prognostic value for transition to psychosis. Importantly, the analyses on the PNC dataset did not support the same pattern of association in subclinical psychiatric conditions other than psychosis. The specificity of these findings should be confirmed by further work, as it is a requirement for a potential marker of psychosis risk occurring before the onset of a chronic diagnosis. For this brain‐behavior relationship to be considered an actual marker of psychosis, further evidence is required.^[^ ^54^ ^]^ Indeed, the retrospective nature of our data intrinsically limits the full translation into an actual biomarker, which may require a preregistered analysis with clear clinical inclusion criteria, and the use of the same neuroimaging sequence and neuropsychological test administration across multiple sites. However, the current study represents one prerequisite for future efforts to consider the brain‐behavior relationship we identified as a candidate biomarker.

Future studies should also examine the approach proposed in this paper in conjunction with additional graph‐based methods for investigating functional brain connectivity. Besides large‐scale connectivity, which was employed in this study, it may be beneficial to consider graph metrics focused on connectivity within the sub‐networks, for example, intrinsic connectivity.^[^ ^55^ ^]^ Intrinsic connectivity is reported to be altered in individuals with psychosis.^[^ ^55^, ^56^ ^]^ More in general, our work suggests that graph measures during resting state could potentially represent sensitive measures to characterize psychosis.

The findings of the present study come with limitations. Cognitive and MRI measures may have been collected on different days. Insufficient test‐retest reliability of the neuropsychological and resting state measures may have masked further findings.^[^ ^57^ ^]^ This very feature is also a strength of these findings, however, considering that the effects are not zeroed by testing time mismatches. Perhaps most importantly, the difference between the neuropsychological instruments employed to evaluate executive function across cohorts could be construed as a limitation. It certainly reduces the replication chances. For the same reason, however, these findings rule out instrument specificity. We suggest the presence of a broader relationship between executive function and the connectivity of right prefrontal‐striatal circuits. Finally, we have no evidence regarding the heritability and association of this candidate marker with familial and genetic risk for psychosis or schizophrenia, considering the negative results in SIBs.

## 4 Conclusion

The results support the idea of a functional role of the prefrontal‐striatal circuitry in NC that is altered in psychosis. We contend that some salient features of psychosis, such as alterations in brain or in cognitive function, may be more relevant in the context of brain‐behavior coupling than on their own right. We conclude that the right prefrontal‐striatal circuit we identified shows alterations in chronic psychosis that extend into the early phase of the disorder and may be further investigated as a potential biomarker of psychosis risk.

## 5 Experimental Section

### Participants

This study consists of two phases: the main investigation and external replication, involving a total of 1,203 individuals. For the main investigation, 269 individuals were enrolled in 4 independent samples: 117 NC in a discovery cohort, 87 NC in an internal replication cohort, 43 chronic patients with psychosis (PSY; 24 patients with schizophrenia and 19 with bipolar disorder with psychosis), and 22 individuals with STPS. Sample demographics are shown in **Table** **1**.

**Table 1:** Demographic and cognitive characteristics of the cohorts included in this study.

Phase	Cohort	Sample size (male)	Age (mean ± SD)	WCST‐TE (mean ± SD)	NB‐EI (mean ± SD)	WMS‐TS (mean ± SD)	TMT B‐A (mean ± SD)	CPT‐FA (mean ± SD)
**Main investigation UNIBA**	MI‐NCD	117 (57)	26.36 ± 6.96	23.66 ± 17.99	0.17 ± 0.10	101.79 ± 9.22	27.34 ± 18.53	0.05 ± 0.04
	MI‐NCR	87 (42)	25.60 ± 5.06	16.83 ± 10.82	0.14 ± 0.09	105.29 ± 13.36	26.35 ± 17.52	0.05 ± 0.06
	MI‐PSY	43 (32)	32.74 ± 10.15	41.02 ± 27.41	0.07 ± 0.06	88.51 ± 17.96	57.58 ± 38.03	0.10 ± 0.16
	MI‐STPS	22 (9)	20.64 ± 4.20	25.36 ± 17.89	0.09 ± 0.05	92.72 ± 21.75	60.18 ± 48.64	0.10 ± 0.11
	TOTAL‐UNIBA	269 (140)	26.67 ± 7.48	24.36 ± 19.59	0.14 ± 0.10	100.07 ± 14.74	34.54 ± 29.03	0.06 ± 0.08
**External replication PNC**	PNC‐TD	57 (34)	18.07 ± 1.68	PCET‐IR 0.25 ± 0.16	–	–	–	–
	PNC‐STPS	45 (17)	17.71 ± 1.52	PCET‐IR 0.28 ± 0.14	–	–	–	–
	PNC‐OD	229 (95)	18.29 ± 1.76	PCET‐IR 0.28 ± 0.16	–	–	–	–
	TOTAL‐PNC	331 (146)	18.17 ± 1.72	PCET‐IR 0.27 ± 0.16	–	–	–	–
**External replication PRONIA**	PRONIA‐NC	202 (72)	25.39 ± 5.86	DSST‐CM 66.75 ± 9.59	–	–	–	–
	PRONIA‐PSY	80 (36)	26.23 ± 6.21	DSST‐CM 52.16 ± 12.63	–	–	–	–
	PRONIA‐STPS	89 (43)	23.92 ± 5.00	DSST‐CM 58.87 ± 10.90	–	–	–	–
	TOTAL‐PRONIA	371 (151)	25.22 ± 5.79	DSST‐CM 61.71 ± 12.16	–	–	–	–
**External replication LIBD**	LIBD‐NC	149 (94)	29.14 ± 8.85	WCST‐TE 14.25 ± 7.68	–	–	–	–
	LIBD‐PSY	54 (34)	28.17 ± 7.56	WCST‐TE 30.61 ± 23.63	–	–	–	–
	LIBD‐SIB	29 (15)	30.93 ± 11.21	WCST‐TE 11.48 ± 5.08	–	–	–	–
	TOTAL‐LIBD	232 (143)	29.14 ± 8.89	WCST‐TE 17.71 ± 14.85	–	–	–	–

Abbreviations: MI‐NCD: Neurotypical controls in discovery cohort of the main investigation; MI‐NCR: Neurotypical controls in within‐site replication cohort of the main investigation; MI‐PSY: Patients with psychosis of the main investigation; MI‐STPS: Individuals with subthreshold psychotic symptoms of the main investigation; PNC: Philadelphia Neurodevelopmental Cohort; PNC‐TD: Individuals with typical development; PNC‐STPS: Individuals with a developmental trajectory toward psychotic disorders; PNC‐OD: Individuals with a developmental trajectory toward other psychiatric disorders; PRONIA: Personalised Prognostic Tools for Early Psychosis Management dataset; PRONIA‐NC: Neurotypical controls in PRONIA dataset; PRONIA‐PSY: Patients with psychosis in PRONIA dataset; PRONIA‐STPS: Individuals with subthreshold psychotic symptoms in PRONIA dataset; LIBD: Lieber Institute for Brain Development; LIBD‐NC: Neurotypical controls in LIBD dataset; LIBD‐PSY: Patients with psychosis in LIBD dataset; LIBD‐SIB: Unaffected siblings of patients with schizophrenia in LIBD dataset; SD: Standard deviation; WCST‐TE: Number of total errors at Wisconsin Card Sorting Test; NB‐EI: N‐Back efficiency index; WMS‐TS: Wechsler Memory Scale – Total score; TMT B‐A: Trail Making Test – difference between Part B and part A; CPT‐FA: Continuous Performance Test – False alarms ratio; PCET‐IR: Proportion of incorrect responses at Penn Conditional Exclusion Test; DSST‐CM: Number of correct symbol‐number matching at Digit Symbol Substitution Test. Please note that in PNC and PRONIA replication samples, WCST‐TE is replaced by PCET‐IR and DSST‐CM, respectively.

In the external replication phase, the PNC dataset, the multi‐site PRONIA dataset, and the LIBD dataset were used. In PNC, participants were differentiated based on different developmental trajectories (Section S1.1, Supporting Information), deriving 57 individuals with typical development (PNC‐TD), 45 individuals with a developmental trajectory toward psychotic disorders (PNC‐STPS), and 229 with a developmental trajectory toward other psychiatric disorders (PNC‐OD). From the PRONIA dataset 202 NC (PRONIA‐NC) were included, 89 individuals who met the criteria for the STPS state (PRONIA‐STPS), and 80 patients with recent‐onset psychosis (PRONIA‐PSY). From the LIBD dataset 149 NC (LIBD‐NC) were included, 54 patients with schizophrenia (LIBD‐PSY), and 29 unaffected siblings of patients with schizophrenia (LIBD‐SIB). Group classification criteria as well as inclusion and exclusion criteria are described in Section S1.1 (Supporting Information). Potential differences in the distribution of clinical symptoms among groups consisting of individuals with STPS are reported in Section S1.6 (Supporting Information).

### Ethical Statement

The study was approved by the local Independent Ethics Committee of Azienda Ospedaliero‐Universitaria Consorziale Policlinico di Bari (Project number: 4754). The written informed consents were obtained from all the participants.

### Neuropsychological Assessment

In the main investigation, a battery of neuropsychological tests was used focusing on cognitive functions critically impaired in psychotic disorders, such as executive function, attention, information processing speed, and working memory (Table 1).^[^ ^58^ ^]^ More specifically, a set of cognitive functioning indices was derived previously found to be associated with psychosis,^[^ ^58^, ^59^ ^]^ such as: the total error number (WCST‐TE) at the Wisconsin Card Sorting Test (WCST), the N‐Back efficiency index (NB‐EI, i.e., the quotient between accuracy and RT) from the N‐Back task, the total score of the Wechsler Memory Scale (WMS‐TS), the differential score between the part A and the part B of the Trail Making Test (TMT‐BA) and the false alarm rate at the Continuous Performance Task (CPT‐FA).

In the replication phase, the same or comparable cognitive tests were used as those employed in the investigation phase. In particular, the WCST‐TE was considered in the LIBD dataset, while the WCST was replaced with the Penn Conditional Exclusion Test, calculating the proportion of incorrect responses (PCET‐IR), in the PNC dataset. Similarly, in the PRONIA dataset, the Digit Symbol Substitution Test was considered, calculating the inverse number of correct symbol‐number correspondences (DSST‐ICM). Full descriptions of the neuropsychological tests used and details about the calculation of indices can be found in Section S1.2 (Supporting Information).

### Statistical Analysis

*Graph‐Based Network Analysis and Meta‐ROI Identification*. To carry out the graph‐based functional connectivity analysis, each participant underwent structural magnetic resonance imaging (sMRI) and resting state‐based functional magnetic resonance imaging (rs‐fMRI) on a 3 Tesla scanner (Table S1, Supporting Information). The MRI images were preprocessed using standard procedures, described in detail in Section S1.3 (Supporting Information)

The analysis of graph‐based functional connectivity measures was first carried out in non‐clinical samples (both NC cohorts of the main investigation) to identify neurotypical brain‐behavior patterns. To estimate centrality measures, This investigation was focused on a set of 160 distinct cortical, subcortical, and cerebellar ROIs, identified by the Dosenbach atlas. This atlas was generated based on a meta‐analysis of task‐related fMRI data. It was considered more reliable for the construction of functional connectivity networks than anatomical atlases^[^ ^60^ ^]^ and had been extensively used in rs‐fMRI experiments.^[^ ^61^ ^]^ Then, for each ROI, two global graph‐based centrality metrics were calculated, namely the degree centrality and the betweenness centrality, which provide different and complementary information about the structure and functionality of the graph and were easily interpretable. Details about the ROIs selection and functional connectivity methods are reported in Section S1.4 (Supporting Information).

**Figure 5:**
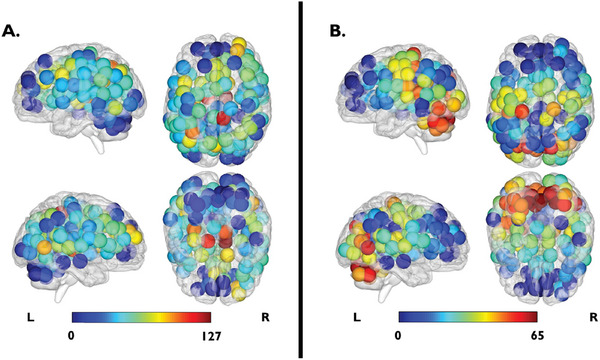
Degree and betweenness centrality mean values for each Dosenbach's ROIs within the MI‐NCD cohort. A) Panel shows the average betweenness centrality value of each ROI. B) Panel shows the average degree centrality value of each ROI. The color bars indicate the betweenness and degree of each ROI in the whole brain. Two lateral and two axial views are showed. Abbreviations: MI‐NCD: Neurotypical controls in discovery cohort of the main investigation (*n = 117*); L: Left; R: Right.

First, the two measures of centrality (betweenness centrality and degree centrality) were calculated in each Dosenbach ROI and for each NC subject in the discovery cohort (*n = 117*). Subsequently, the mean values of betweenness centrality and degree centrality were calculated for each ROI among the subjects in the cohort. The spatial correlation structure of these metrics was analyzed across contiguous regions, as signal correlation and functional relatedness between nearby regions may justify reducing the dimensionality of the whole‐brain network to several local networks.^[^ ^62^ ^]^ To explore this possibility, the degree centrality and betweenness centrality mean values were plotted within the NC discovery cohort on a brain template, using the BrainNet Viewer toolbox.^[^ ^63^ ^]^ Indeed, a clustered distribution of the centrality measures was observed (**Figure** **5**): neighboring ROIs had similar mean centrality values throughout the brain, effectively forming spatial clusters of centralities. Accordingly, it was chose to cluster the ROIs based on into functional similarity and spatial proximity, therefore generating “meta‐ROIs”. This procedure was performed through hierarchical Ward clustering^[^ ^39^ ^]^ analysis fed with the coordinates of the ROIs (x, y, z according to the MNI atlas) and the mean values of the ROI‐betweenness centrality and ROI‐degree centrality previously calculated in NC subjects using SPSS (IBM Corp. Released 2011). This clustering approach was chose to obtain homogeneous clusters since this method minimizes the within‐cluster variance.^[^ ^64^ ^]^ The analyses were performed separately for each hemisphere to minimize interhemispheric differences in structural and functional connectivity.^[^ ^65^ ^]^ To validate the main results using an alternative anatomical atlas, the same procedure was employed for the 90 ROIs of the AAL atlas.^[^ ^41^ ^]^ The detailed description of the procedure is reported in the Section S2.1 (Supporting Information).

*Identification of Neurotypical Pattern of Brain‐Behavior Association in the Main Investigation*. In the main investigation, it was first pursued the goal of identifying neurotypical patterns of association between graph‐based centrality measures and measures of cognition in these samples of NC. To this aim, the centrality measures of the meta‐ROIs was summarized by averaging the betweenness centrality and degree centrality values of each ROI included in the meta‐ROI. Then, an outlier detection procedure was performed on the following variables: age, neuropsychological scores, and meta‐ROI centrality measures (betweenness centrality and degree centrality). Values were excluded beyond three times the interquartile range. Then, two‐side Pearson's partial correlation analyses was used to test associations between meta‐ROI measures of centrality (betweenness centrality – degree centrality) and neuropsychological scores (*P *< 0.05, False Discovery Rate – FDR – corrected)^[^ ^66^ ^]^ in the NC discovery cohort (*n = 117*), covarying for age and sex. The FDR correction was applied separately for betweenness centrality and degree centrality analyses. Results were corrected for multiple comparisons performed on the 25 meta‐ROIs for five neuropsychological variables, i.e., 25 × 5 = 125 partial correlations for betweenness centrality, and 125 for degree centrality.

To identify any replicable association between centrality measures and neuropsychological scores, the meta‐ROI betweenness centrality and degree centrality measures were computed in the NC replication cohort (*n = 87*; using the same ROI clustering of the NC discovery cohort) and the one‐side Pearson's partial correlation analyses was replicated between meta‐ROI betweenness centrality and degree centrality values and neuropsychological scores, using age, sex and the MRI parameter of repetition time (TR) as covariates (*P* < 0.05). Finally, to test for differences in the two neurotypical cohorts (NC discovery and NC replication), Fisher's transformation was used from r to z (*P* < 0.05) on replicable correlations.^[^ ^67^ ^]^ The meta‐ROIs showing a replicable brain‐behavior association were compared with literature evidence using NeuroMark^[^ ^68^ ^]^ (see Sections S1.5 and S2.2, Supporting Information). Also, in both NC cohorts (*n = 117; n = 87*), the potential sex‐related differences in the association between the centrality measures of replicable meta‐ROIs and cognitive performance were investigated (Section S1.7, Supporting Information). The values of the neuropsychological scores (mean ± SD) for both NC cohorts were reported in Table 1.

*Alteration of Brain‐Behavior Association in Clinical Populations of the Main Investigation*. Then the second aim of the main investigation was pursued: to test whether the neurotypical patterns of brain‐behavior association between centrality measures and cognitive scores could be affected in PSY (*n = 43*) and in individuals with STPS (*n = 22*). For this purpose, the centrality measures of meta‐ROIs was calculated that showed a replicable brain‐behavior association in the two samples of NC, and for these, a two‐side Pearson's partial correlation were computed with neuropsychological scores in both the clinical cohorts (PSY and individuals with STPS) using age, sex, and TR as covariates (*P* < 0.05). Using Fisher's r to z transformation any differences between PSY and individuals were evaluated with STPS and between them and NC (*P* < 0.05).^[^ ^67^ ^]^ Finally, in both cohorts, the potential sex‐related differences in the association between the centrality measures of replicable meta‐ROIs and the neuropsychological scores were investigated (Section S1.7, Supporting Information). The neuropsychological scores (mean ± SD) for both the clinical cohorts are detailed in Table 1.

*Investigations on Centrality Measure and Cognitive Performance in the Main Investigation*. It was focused on cognitive function and meta‐ROI centrality measures that showed an altered brain‐behavior association in the clinical samples performing, on these variables, additional analyses to explore any pre‐existing differences between the cohorts. By two‐side ANOVA (*P* < 0.05), any differences in centrality measures were assessed among NC replication cohort (*n = 87*), PSY (*n = 43*), and STPS subjects (*n = 22*). Another two‐side ANOVA was performed on neuropsychological scores (*P* < 0.05) among the three cohorts to explore any effects attributable to differences at the cognitive level.

*External Replication Phase*. In the external replication phase, the replicability of the associations found in the internal cohorts was studied in each PNC group. Centrality values were extracted from the meta‐ROIs that showed replicable association with cognitive measures for each of the three developmental cohorts (PNC‐TD, *n = 57*; PNC‐STPS, *n = 45*; PNC‐OD, *n = 229*). Then, in each cohort, a Pearson's partial correlation (one‐side for PNC‐TD and PNC‐STPS; two‐side for PNC‐OD) was calculated between the centrality measure of the meta‐ROIs identified in the main investigation and the PCET‐IR score (*P* < 0.05), using age and sex as covariates. Potential differences in centrality values or cognitive performance between the PNC‐TD and PNC‐STPS cohorts were explored by two‐sample t‐test (*P* < 0.05).

Then, the replicability of the associations found in the main investigation was studied in each PRONIA group. Once again, centrality values were extracted from the meta‐ROIs that showed replicable association with cognitive measures for each of the three cohorts (PRONIA‐NC, *n = 202*; PRONIA‐PSY, *n = 80*; PRONIA‐STPS, *n = 89*). It was then calculated, in each group, the one‐side partial Pearson correlation between the meta‐ROI centrality measure identified in the main survey and the DSST‐ICM score (*P* < 0.05), using age, sex, and MR scanner as covariates. Through two‐sample t‐tests (*P* < 0.05), potential differences were investigated in centrality values or cognitive performance, among the cohorts that showed a significant association between brain and behavior.

Finally, in each LIBD group the replicability of the associations discovered in the main investigation was examined. Once again, centrality values were derived from the meta‐ROIs that exhibited replicable association with cognitive measures for the three cohorts (LIBD‐NC, *n = 149*; LIBD‐PSY, *n = 54*; LIBD‐SIB, *n = 29*). Subsequently, within each group, the partial Pearson correlation (one‐side for LIBD‐NC and LIBD‐PSY; two‐side for LIBD‐SIB) was computed between the meta‐ROI centrality measure identified in the main survey and the WCST‐TE score (*P* < 0.05), controlling for age and sex. Using two‐sample t‐tests (*P* < 0.05), potential differences were explored in centrality values or cognitive performance among the cohorts that showed a significant brain‐behavior association. In all cohorts of the external replication, potential sex‐related differences were investigated in the association between the degree centrality of the replicable meta‐ROIs and the cognitive performance (Section S1.7, Supporting Information). The neuropsychological scores (mean ± SD) for all cohorts of the external replication phase are reported in Table 1.

## Conflict of Interest

A.B. received consulting fees from Biogen and lecture fees from Otsuka, Janssen, and Lundbeck. G.B. reported receiving personal fees from Lundbeck outside the submitted work. N.K. received honoraria for talks presented at education meetings organized by Otsuka/Lundbeck. D.R.W. serves on the Scientific Advisory Boards of Sage Therapeutics and Pasithea Therapeutics. G.P. received lecture fees from Lundbeck. All other authors report no biomedical financial interests or potential conflicts of interest. The funding organizations stated above were not involved in the design and realization of the study; the collection, management, analysis, and interpretation of the data; the preparation, review, or approval of the manuscript; and decision to submit the manuscript for publication.

## Author Contributions

L.F. and G.S. contributed equally to this work. L.F. performed data curation, investigation, methodology, validation, visualization, wrote the original draft, reviewed, and edited the draft. G.S. performed data curation, formal analysis, investigation, methodology, validation, visualization, wrote the original draft, reviewed, and edited the draft. R.P. performed data curation, methodology, wrote, reviewed, and edited the draft. A.T. performed data curation, wrote, reviewed, and edited the draft. G.B., N.K., and A.R. performed funding acquisition, wrote, reviewed, and edited the draft, and provided resources. M.O.B., A.L.G., S.S.H., M.N., A.R., P.S., W.U., A.N.R.P., and P.C. performed data curation, wrote, reviewed, and edited the draft. L.K.‐I. performed data curation, wrote, reviewed, and edited the draft. T.P. provided resources and wrote, reviewed, and edited the draft. F.S. performed methodology, wrote, reviewed, and edited the draft. D.R.W. provided resources and wrote, reviewed, and edited the draft. A.B. performed funding acquisition, wrote, reviewed, and edited the draft, and provided resources. L.A.A. performed investigation, methodology, validation, and wrote the original draft. G.P. performed conceptualization, funding acquisition, investigation, methodology, supervision, provided resources, wrote the original draft, reviewed, and edited the draft.

## Supporting information



Supporting Information

## Data Availability

European rules on the treatment of confidential research data require contacting each participant before sharing their data with entities not explicitly approved in the informed consent. For this reason, raw data supporting the findings of this study cannot be shared for the UNIBA dataset. Upon reasonable request, data can be accessed for analyses without sharing them. A data access request can be sent to giulio.pergola@uniba.it. The individual de‐identified raw data from the LIBD analyzed in this study are available from the author, D.R. Weinberger (drweinberger@libd.org) upon reasonable request. PNC data are publicly available via eRA Commons. The full URL to obtain more details and submit the access request is https://www.ncbi.nlm.nih.gov/projects/gap/cgi‐bin/study.cgi?study_id=phs000607.v3.p2.PRONIA data are not publicly available due to Institutional Review Board restrictions ‐ since the participants did not consent to public availability. Prof. N. Koutsouleris had full access to PRONIA data and takes responsibility for their integrity (nikolaos.koutsouleris@med.uni‐muenchen.de).
