# Supplementary material for: Altered Brain‐Behavior Association During Resting State is a Potential Psychosis Risk Marker
Source: Adv Sci (Weinh). 2025 Apr 2;12(26):2405700. doi: 10.1002/advs.202405700 (PMC12245128; doi:10.1002/advs.202405700)
Supplement: Supplementary file 1 — Supporting Information [file ADVS-12-2405700-s001.pdf]

ADVANCED SCIENCE
Open Access

Supporting Information

for *Adv. Sci.*, DOI 10.1002/advs.202405700

Altered Brain-Behavior Association During Resting State is a Potential Psychosis Risk Marker

Leonardo Fazio, Giuseppe Stolfa, Roberta Passiatore, Angelantonio Tavella, Giuseppe Blasi, Madalina O. Buciuman, Aaron L. Goldman, Shalaila S. Haas, Lana Kambeitz-Ilankovic, Nikolaos Koutsouleris, Monica Nicoli, Teresa Popolizio, Antonio Rampino, Anne Ruef, Fabio Sambataro, Pierluigi Selvaggi, William Ulrich, Daniel R. Weinberger, Apulian Network on Risk for Psychosis, PRONIA Consortium, Alessandro Bertolino, Linda A. Antonucci and Giulio Pergola**

Supporting Information

Altered Brain-Behavior Association During Resting State Is a Potential Psychosis Risk Marker

Leonardo Fazio,[†] Giuseppe Stolfa,[†] Roberta Passiatore, Angelantonio Tavella, Giuseppe Blasi, Madalina O. Buciuman, Aaron L. Goldman, Shalaila S. Haas, Lana Kambeitz-Ilankovic, Nikolaos Koutsouleris, Monica Nicoli, Teresa Popolizio, Antonio Rampino, Anne Ruef, Fabio Sambataro, Pierluigi Selvaggi, William Ulrich, Daniel R. Weinberger, Apulian Network on Risk for Psychosis, PRONIA Consortium, Alessandro Bertolino, Linda A. Antonucci[*], and Giulio Pergola[*]

[†]These authors contributed equally to this work.
*Corresponding authors

Section 1: Supplementary Information

Section 1.1: Participants, Groups Classification Criteria and Inclusion/Exclusion Criteria

For the main investigation participants, the main diagnosis of schizophrenia and bipolar disorder was ascertained through the Structured Clinical Interview for DSM-IV[1] (SCID). The subthreshold psychotic symptoms (STPS) condition was defined by: (1) cognitive impairment, as assessed by the Schizophrenia Proneness Instrument[2] (SPI-A), and/or (2) very high risk criteria for psychosis, according to the Structured Interview for Psychosis-Risk Syndromes[3] (SIPS) criteria as reported in previous papers.[4] Exclusion criteria were: age below 16 years, moderate or severe medical conditions, acute substance use, and any contraindication to MRI. For the two neurotypical control (NC) cohorts, we also excluded individuals with 1st-degree familiarity for psychotic disorders, current use of medications with central nervous system effects, and personal history of psychiatric disorders. All patients with psychosis (PSY) were pharmacologically stable and had been on antipsychotic treatment for at least 1 month. The individuals with STPS were excluded when they had any antipsychotic medication intake in the past 3 months before enrollment in the study or for more than 30 cumulative days.

For the external replication, we used the Philadelphia Neurodevelopmental Cohort (PNC) dataset, the multi-site PRONIA (Personalised Prognostic Tools for Early Psychosis

Management) dataset and the NIH SIB-Study dataset available at the Lieber Institute for Brain Development (LIBD). From PNC, we divided individuals into 3 cohorts based on different developmental trajectories. These trajectories were estimated using a computerized interview (GOASSESS), which explores a broad set of symptoms covering the major psychiatric disorders described in the DSM-IV-TR.[5] Specifically, the GOASSESS includes 113 items corresponding to the symptoms of 14 psychopathological domains, as reported in a previous study.[6] Participants who exhibited one or more symptoms associated with at least moderate impairment or distress in the psychopathological domain of psychosis were identified as individuals with a developmental trajectory toward psychotic disorders (PNC-STPS). In contrast, individuals who showed one or more symptoms associated with at least moderate impairment or distress in any psychopathological domain except psychosis were identified as individuals with a developmental trajectory toward other psychiatric disorders (PNC-OD). Finally, participants who showed no symptoms associated with at least moderate impairment or distress were defined as at typical development (PNC-TD). As a result, we had 57 PNC-TD, 45 PNC-STPS, and 229 PNC-OD (Table 1). The PRONIA dataset included multimodal data from neurotypical control participants (PRONIA-NC) and young patients who meet criteria for the STPS condition (PRONIA-STPS) or recent-onset psychosis (PRONIA-PSY) from different European countries.[7] Specifically, we used clinical, cognitive and imaging data of 371 individuals divided in 3 cohorts: 202 PRONIA-NC, 80 PRONIA-PSY and 89 PRONIA-STPS. Subjects included in this study were recruited from five European countries, and their imaging data were acquired using nine different MRI scanners. The LIBD dataset included multimodal data from neurotypical control participants (LIBD-NC), patients with diagnosis of schizophrenia (LIBD-PSY) and 29 unaffected siblings of patients with schizophrenia (LIBD-SIB). Specifically, we used cognitive and imaging data of 232 individuals divided in 3 cohorts: 149 LIBD-NC, 54 LIBD-PSY and 29 LIBD-SIB. The LIBD-NC cohort was established using the male-to-female ratio LIBD-PSY cohort as a reference. Specifically, propensity score matching[8] was initially performed on age between males and females in the LIBD-NC cohort. Subsequently, from the matched group of females, 55 subjects were randomly selected. The SIB condition was defined by the absence of DSM IV Axis I diagnoses, with the contemporary presence of a first-degree relative affected by a DSM IV Axis I diagnosis. As in the main survey, we excluded individuals younger than 16 years, with moderate or severe medical conditions, and with acute substance use in the external replication.

Section 1.2: Neuropsychological Assessment: Descriptions of Neuropsychological Tests and Cognitive Indices

For the neuropsychological assessment, we used a battery of tests focusing on impaired cognitive function in psychosis, from which we derived indices of cognitive functioning. The battery included the Wisconsin Card Sorting Test, the N-back task, the Wechsler Memory Scale, the Trail Making Test, and the Continuous Performance Test – AX.

In detail, the Wisconsin Card Sorting Test[9] is a test used to assess several frontal lobe functions such as strategic planning, use of environmental feedback to achieve a goal and modulation of impulsive responses. The test uses stimulus and response cards characterized by three criteria (shapes, colors, and numbers) with four levels each. Participants are instructed to match the cards without knowing which criteria to follow. The only feedback provided is the correctness of the matching. As the test progresses, there are unannounced shifts in the matching principle that require the individual to change their strategy. We recorded the number of total errors done (WCST-TE).[10]

The N-back task is a working memory task.[11] The stimuli are numbers (1-4) shown in a random sequence and displayed at the corners of a diamond-shaped box. The task has a non-memory-driven control condition (0-Back) that requires individuals to identify the currently viewed stimulus. In another condition, working memory is assessed as the participant must recall a stimulus seen two stimuli earlier (2-Back) while continuing to encode other incoming stimuli. Performance data were recorded as the percentage of correct responses (accuracy) and reaction time (RT) expressed in milliseconds. We calculated the N-Back efficiency index (NB-EI), i.e., the quotient between accuracy and RT.[12]

The Wechsler Memory Scale[13] is a battery measuring many memory functions such as orientation in time and place, mental control, logical memory, visual reproduction, and associated learning. The WMS total score (WMS-TS) was used here as in previous studies.[14]

The Trail Making Test[15] assesses attention, visuospatial coordination, and cognitive flexibility. The test includes two parts: part A, in which the targets are all numbers and the test taker must connect them in sequential order; and part B, in which the participant alternates between numbers and letters. The goal of the test is to finish part A and part B as quickly as possible. The difference in the time taken to complete the two parts (TMT-BA) is considered an indicator of executive functioning and cognitive flexibility and was used here as in previous studies.[14]

The Continuous Performance Test – AX,[16] examines the attentional domain, specifically sustained and selective attention. During the task, single letters were presented sequentially on the screen and participants responded when they saw an X after an A. Randomly, a non-cue

letter could precede the target (e.g., B-X), or a distractor could follow a cue (e.g., A-Y), or a non-cue letter could precede a distractor (e.g., B-Y). We calculated the ratio of the false alarms, i.e. the inappropriate responses given to a distractor appearing after the letter "A" (CPT-FA).[14] In the replication phase, we used the same or comparable cognitive tests as those employed in the investigation phase. In the PNC dataset, we replaced the Wisconsin Card Sorting Test, with the Penn Conditional Exclusion Test (Table 1). The Penn Conditional Exclusion Test measures mental flexibility and is comparable to the Wisconsin Card Sorting Test in terms of task structure and the dimensions assessed.[17] Participants decided which of 4 objects did not belong to the other 3 based on one of three sorting criteria (shape, size, and line thickness). Sorting principles changed after ten successive correct responses, and participants used feedback to guide criterion discovery and change response strategy. The proportion of incorrect responses (PCET-IR) was included in our analyses, similar to the main survey (total incorrect responses were unavailable in the PNC dataset). In the PRONIA dataset, instead, we replaced the Wisconsin Card Sorting Test with the Digit Symbol Substitution Test (Table 1). This test, requiring matching symbols and numbers based on a key located at the top of the page,[18] involves different executive functions, such as of cognitive flexibility and set-shifting, as the Wisconsin Card Sorting Test. For this test, we considered the inverse number of correct symbol-number correspondences (DSST-ICM). We chose to reverse the score in order to keep the same direction as the other executive functions indices we used. In the LIBD dataset, we considered the WCST-TE (Table 1) as in the investigation phase.

Section 1.3: MRI Data Acquisition and Preprocessing

MRI assessments were carried out at thirteen different 3 Tesla scanners (two for the main investigation, one in the PNC dataset, nine in the PRONIA dataset and one in the LIBD dataset – details in Table S1). We collected a structural MRI (sMRI) and a resting state-based functional MRI (rs-fMRI) for each participant. For the sMRI, we used T1-weighted structural images with either MPRAGE or T1-FFE sequences. The specific parameters for each dataset of each acquired sMRI scan were reported in Table S1. The structural images were processed with the Computational Anatomy Toolbox 12 (CAT12, Structural Brain Mapping group, Jena University Hospital, Jena, Germany - http://www.neuro.uni-jena.de/cat12/) included in SPM12 (Statistical Parametric Mapping, Institute of Neurology, London, UK - https://www.fil.ion.ucl.ac.uk/spm). T1-weighted images were normalized to a standard brain (MNI152) using a diffeomorphic recording algorithm (DARTEL[19]) and segmented into different tissue classes (gray matter, white matter and cerebrospinal fluid) based on probability

maps. All images were then modulated through Jacobian determinants to preserve the initial volumes and were smoothed with a 3 mm isotropic Gaussian filter. We excluded the raw images with technical artifacts like blurring, ringing, wrapping and incomplete head coverage,[20] and the segmented ones with excessive noise, poor contrast and/or poor boundaries.[21]

During each rs-fMRI scan across all datasets involved, participants were instructed to keep their eyes open and remain still. In the UNIBA, PNC and LIBD dataset, participants were instructed to remain still, awake, with their eyes open, and to focus on the crosshair at the center of a white screen.[22] In the PRONIA dataset, while acquiring rs-fMRI scans, subjects were instructed to keep their eyes open and not to think about anything.[23] Scans were acquired using the gradient-echo echo-planar imaging (GE-EPI) sequence. The specific parameters for each dataset of each rs-fMRI acquired scan were reported in Table S1. Notably, in the NC-R, PSY and STPS cohorts of the main investigation, rs-fMRI scans have been acquired using two different sequences. We accounted for this difference in the following correlation analysis, specifying repetition time (TR) as covariate. In the three PNC cohorts, the first 4 volumes were removed from the time series to allow for signal stabilization, as was done in a previous study.[24] This procedure was embedded in the scanner sequence for the other scanners we used. In the PRONIA cohorts, rs-fMRI scans have been acquired using nine different MR scanner. We accounted for this difference in the following correlation analysis, specifying MR scanner as covariate.

We used SPM12 for the neuroimaging analysis (www.fil.ion.ucl.ac.uk/spm). For each participant, the volumes were realigned to correct for head movement, and the motion parameters were obtained.[25] The realigned images were resized, co-registered to T1-weighted structural images, spatially normalized into a standard space (MNI 152), and smoothed with a 6 mm FWHM isotropic kernel. Finally, all images were denoised through Wavelet Despike.[26]

Quality-based inclusion criteria were: good quality structural images, no scanning artifacts, low movement (translation < 3 mm, rotation < 3°, Framewise Displacement variation across volumes – FD<0.05.[27] In the PRONIA dataset, since motion parameters were not available for all participants, we chose to use a stronger global threshold to include subjects, i.e. FD<0.2. We excluded the images that did not meet the quality inclusion criteria, and the remaining ones were used to investigate functional connectivity through graph-based network analysis.

Section 1.4: Graph-Based Network Analysis

The analysis of graph-based FC measures was first carried out in non-clinical samples (both NC cohorts of the main investigation) to identify normative brain-behavior patterns. To estimate centrality measures, we focused our investigation on a set of 160 distinct cortical,

subcortical, and cerebellar ROIs, identified by the Dosenbach atlas.[28] This atlas was generated based on a meta-analysis of task-related fMRI data. It is considered more reliable for the construction of FC networks than anatomical atlases[29] and has been extensively used in rs-fMRI experiments.[30]

We then created a set of 10 mm spheres centered on the MNI coordinates of each of the 160 ROIs, using the MarsBar toolbox (http://marsbar.sourceforge.net) as previously done.[30b] To minimize potential differences in brain coverage between cohorts, we calculated the number of the acquired voxels within each ROI, and we excluded those ROI including below 80% of the voxels acquired in the group of participants.[31] In this way, we selected 144 ROIs with optimal coverage, and we extracted from them the average signal using MarsBar toolbox. Then, we computed the ROI-ROI pairwise correlation matrices for each individual.

To identify regions representing potential hubs of brain network organization and information processing,[32] for each of the ROIs we calculated two global graph-based centrality metrics, namely the degree centrality and betweenness centrality. We chose these two metrics as they provide different and complementary information about the structure and functionality of the graph and are easily interpretable.[33] Specifically, degree centrality is defined as the number of node connections, so nodes with high degree centrality are highly connected to the rest of the brain and may thus be particularly influential on the functioning of the network.[34] Betweenness centrality refers to the involvement of nodes in short paths through the network, so nodes with high betweenness centrality are thought to be particularly important for the efficiency of communication between clusters.[32] We calculated the individual values of degree centrality and betweenness centrality for each of the 144 ROIs via GraphVar toolbox.[35] Then, we used the ROI-ROI pairwise correlation matrices to construct a binary graph using a relative thresholding of 0.2 (threshold value equals the percentage of the strongest connections, i.e., a graph was created based on the 20% strongest connections). We excluded the weakest connections in order to reduce noise and to focus on the connections with greatest relevance.[36] We then calculated centrality metrics (betweenness centrality and degree centrality) for each ROI of each subject.

Section 1.5: Spatial Characterization of Replicable Meta-ROIs

To investigate the functional role of meta-ROIs associated with cognitive performance, and to improve the interpretability of the results, we performed a spatial overlap between the ROIs in this study and those already known in the literature. Specifically, the meta-ROIs showing a replicable brain-behavior association with cognitive measures, were overlaid on the 53

NeuroMark components.[37] The NeuroMark components were obtained through an independent component analysis (i.e., an approach different from the one used in this study) and can therefore be an independent reference in interpreting the results. We thus determined which NeuroMark components (and consequently which networks) included regions of meta-ROIs associated with significant patterns of brain-behavior relationship by calculating the R^2 associated with each network.

Section 1.6: Comparison Between Cohorts Consisted of Individuals with Subthreshold Psychotic Symptoms

Potential differences in the distribution of psychopathological symptomatology among the different groups consisted of individuals with subthreshold psychotic symptoms were assessed. Specifically, the prevalent symptomatology in each individual was identified and classified into the following four psychopathological macrodomains into which their respective symptoms converge: psychotic symptoms, anxiety symptoms, manic symptoms, and depressive symptoms. The presence or absence of symptoms was assessed with three different clinical instruments. The Brief Psychiatric Rating Scale was used in the cohort consisted of subjects with STPS of the main investigation: items 1-2 refer to symptoms characterizing anxiety disorders, items 3 to 5 refer to symptoms related to depressive disorders, items 6 to 8 refer to symptoms typical of manic disorders, and items 9 to 15 characterize psychotic disorders. Items with value > 1 indicate the presence of the symptom. A computerized GOASSESS interview was used in the PNC-STPS to assess different developmental trajectories. The GOASSESS includes 113 items corresponding to symptoms of 14 psychopathological domains, as defined in the main text. In the Table S3 are reported subjects who exhibited: - one or more symptoms associated with at least moderate impairment or discomfort in the psychopathological domain of "psychosis spectrum"; - one or more symptoms associated with at least moderate impairment or discomfort in the psychopathological domain of "depression"; - one or more symptoms associated with at least moderate impairment or discomfort in the psychopathological domain of "mania" - one or more symptoms associated with at least moderate impairment or distress in the psychopathological domain of "agoraphobia," "generalized anxiety disorder," "panic disorder," "specific phobias," "post-traumatic disorder," "separation anxiety disorder," and "social anxiety disorder." The Structured Clinical Interview for DSM-IV was used to assess the presence of psychiatric symptoms in PRONIA-STPS. The last item of this interview provided a psychiatric disorder that the subject had or might have had. Specifically, the item is labeled “SCID1_Summary_8_Q01_DiagnosisDroplist” and the clinician is asked to indicate "the

disorder that is (or should be) the primary focus of current clinical attention." Based on this information, subjects were classified and differences in distribution for each psychopathological macrodomain were investigated by Chi-squared ($P < 0.05$) (Supporting Information, Section 2.2 and Table S3).

Section 1.7: Potential Sex-Related Differences in the Brain-Behaviour Association

In each cohort of the main investigation, we investigated potential sex-related differences in the association between the degree centrality of the prefontal-cingulm-striatal meta-ROI and the executive performance. Therefore, in MI-NCD we conducted Pearson's partial correlation analyses to test association between meta-ROI degree centrality and WCST-TE ($P < 0.05$) separately for each sex, controlling for age. Also, in the remaining three cohorts of the main investigation, we conducted Pearson's partial correlation analyses to test association between the meta-ROI degree centrality and the WCST-TE ($P < 0.05$) separately for each sex, controlling for age and TR. In all PNC and LIBD cohorts of the replication phase, we conducted Pearson's partial correlation analyses to test association between the meta-ROI degree centrality and the executive performance ($P < 0.05$) separately for each sex, controlling for age. Finally, in the PRONIA cohorts, we conducted Pearson's partial correlation analyses to test association between the meta-ROI degree centrality and the executive performance ($P < 0.05$) separately for each sex, controlling for age and MRI scanner.

In addition to accounting for biological sex as a factor and assessing the robustness of the main findings using Pearson's partial correlation analyses, we employed general linear models in each study group. These models aimed to examine the main effect of the prefrontal-cingulum-striatal meta-ROI degree centrality on the cognitive performance and to explore the interaction between the sex and the degree centrality on the cognitive performance. Specifically, in the NC discovery cohort, we performed a general linear model (between-subject factor: biological sex; continuous predictor: WCST-TE; covariates: age, and meta-ROI degree centrality - which was associated with the biological sex; $P < 0.05$, two-tailed). In the NC replication cohort, we replicated the general linear model (between-subject factor: biological sex; continuous predictor: WCST-TE; covariates: age, TR, and meta-ROI degree centrality - which was associated with the biological sex; $P < 0.05$, one-tailed). Similarly, we computed a general linear model in each clinical cohort of the main investigation (between-subject factor: biological sex; continuous predictor: WCST-TE; covariates: age, TR, and meta-ROI degree centrality - which was associated with the biological sex; $P < 0.05$, two-tailed). Then, in each PNC cohort, we conducted a general linear model (between-subject factor: biological sex; continuous predictor:

PCET-IR score; covariates: age and meta-ROI degree centrality - which was associated with the biological sex; $P < 0.05$, one-tailed). Additionally, in each PRONIA group, a general linear model (between-subject factor: biological sex; continuous predictor: DSST-ICM score; covariates: age, MR scanner, and meta-ROI degree centrality - which was associated with the biological sex; $P < 0.05$, one-tailed) was conducted. Finally, we computed a general linear model (between-subject factor: biological sex; continuous predictor: WCST-TE score; covariates: age and meta-ROI centrality measure identified in the main survey - which was associated with the biological sex; $P < 0.05$, one-tailed) in each LIBD cohort.

Section 1.8: Additional Brain-Behavior Associations and Differences Across Cohorts

In order to further validate the brain-behavior pattern adopted in this study, we investigated the associations between the degree centrality of the prefrontal-cingulum-striatal meta-ROI and the cognitive scores that previously showed no replicable associations in NC cohorts of the main investigation, both in PSY and in individuals with STPS. To this aim, in the PSY and STPS cohorts, we calculated Pearson's partial correlations between the degree centrality of the meta-ROI and each neuropsychological score other than WCST-TE, namely NB-EI, WMS-TS, TMT-BA and CPT-FA, using age, sex and TR as covariates ($P < 0.05$).

Additionally, we examined the associations between the measures of centrality (betweenness centrality - degree centrality) of the 25 meta-ROIs and all neuropsychological scores in the two clinical cohorts (pFDR<0.05) of the main investigation, using age, sex and TR as covariates. On each cohort, the FDR correction was applied separately for betweenness centrality and degree centrality analyses, multiple-comparison correcting both the 25 meta-ROIs and the five neuropsychological variables, i.e., taking into account 125 partial correlations for betweenness centrality, and 125 for degree centrality. Subsequently, the significant associations were also investigated ($P < 0.05$) in the external cohorts.

We have evaluated group-level differences in whole-brain centrality and in all neuropsychological variables. An ANOVA (pFDR < 0.05) was employed to evaluate the differences in centrality measures (betweenness centrality and degree centrality) of the 25 meta-ROIs among subjects of the main investigation who underwent resting-state scans using the same MRI scanner. This included the replication cohort of neurotypical controls, PSY subjects, and STPS subjects. The FDR correction was applied separately for betweenness centrality and degree centrality analyses, i.e., taking into account 25 ANOVAs for betweenness centrality, and 25 for degree centrality. An additional ANOVA was performed on the five neuropsychological scores (pFDR < 0.05) across the three cohorts to investigate differences at

the cognitive level. The same procedure was conducted on the three external datasets to observed differences (pFDR < 0.05) between cohorts.

Section 2: Supplementary Results

Section 2.1: Validation of Results Using the Automatic Anatomical Labelling Atlas

We have conducted replication analyses using an alternative atlas, the Automatic Anatomical Labelling (AAL) atlas,[38] which includes 90 cortical and subcortical ROIs (45 per hemisphere) and has been extensively used in psychiatric neuroscience.[39] We have replicated the exact analysis pipeline already performed on the Dosenbach atlas.

Specifically, for the AAL atlas, we created 10 mm spheres centered on the MNI coordinates of each of the 90 ROIs using the MarsBar toolbox (http://marsbar.sourceforge.net). To ensure adequate brain coverage, we excluded ROIs with less than 80% of the voxels acquired in the neurotypical controls. This procedure resulted in 73 ROIs with optimal coverage. We then extracted the average signal from these ROIs and computed ROI-ROI pairwise correlation matrices for each individual.

Using the GraphVar toolbox, we constructed a binary graph with a relative threshold of 0.2 and calculated betweenness centrality and degree centrality for each ROI. We performed Ward's hierarchical clustering on the ROI coordinates and the mean betweenness centrality and degree centrality values, resulting in 22 meta-ROIs (AAL-meta-ROIs - 11 per hemisphere). To investigate the replicability of our main findings, we searched for the AAL-based meta-ROI which overlapped the most with the Dosenbach-based prefrontal-cingulum-striatal meta-ROI already reported in the Results of our manuscript. The greatest overlap with this meta-ROI was calculated based on two methods:

1. Spatial Overlap: We calculated the percentage of overlapping voxels between the prefrontal-cingulum-striatal meta-ROI (Dosenbach) and the 11 right hemisphere AAL-meta-ROIs. The highest overlap (16%) was found with a prefrontal-cingular AAL-meta-ROI.
2. Euclidean Distance: We measured the average Euclidean distance between the prefrontal-cingulum-striatal meta-ROI (Dosenbach) and the 11 right hemisphere AAL-meta-ROIs. The minimum distance (19.3 mm) corresponded to the same prefrontal-cingular AAL-meta-ROI identified by the overlap method.

The two approaches concur in identifying a right prefrontal-cingular AAL-meta-ROI, depicted in **Figure S1** below, as the most comparable to the Dosenbach-based meta-ROI utilized in our study. The limited overlap of these meta-ROIs is not surprising, given that the two atlases have an origin based on distinct types of data, which influences the spatial localization of ROIs. The right prefrontal-cingular AAL-meta-ROI included four AAL ROIs, specifically: anterior

cingulate cortex (x,y,z = 8.46, 37.01, 15.84); superior frontal gyrus (x,y,z = 21.90, 31.12, 43.82); middle frontal gyrus (x,y,z = 37.59, 33.06, 34.04), medial superior frontal gyrus (x,y,z = 9.10, 50.84, 30,22).

Therefore, we calculated the mean degree centrality of this AAL-meta-ROI and tested associations with WCST-TE scores with Pearson's partial correlation. In the NC discovery cohort, we found a significant positive correlation (r = 0.16; P = 0.046) between the two variables. In contrast, the PSY and STPS cohorts showed significant negative correlations (r = -0.29; P = 0.033 and r = -0.41; P = 0.043, respectively). These results are in line with those obtained with the Dosenbach atlas and demonstrate that the relationships we have reported in the manuscript generalize to different atlases.

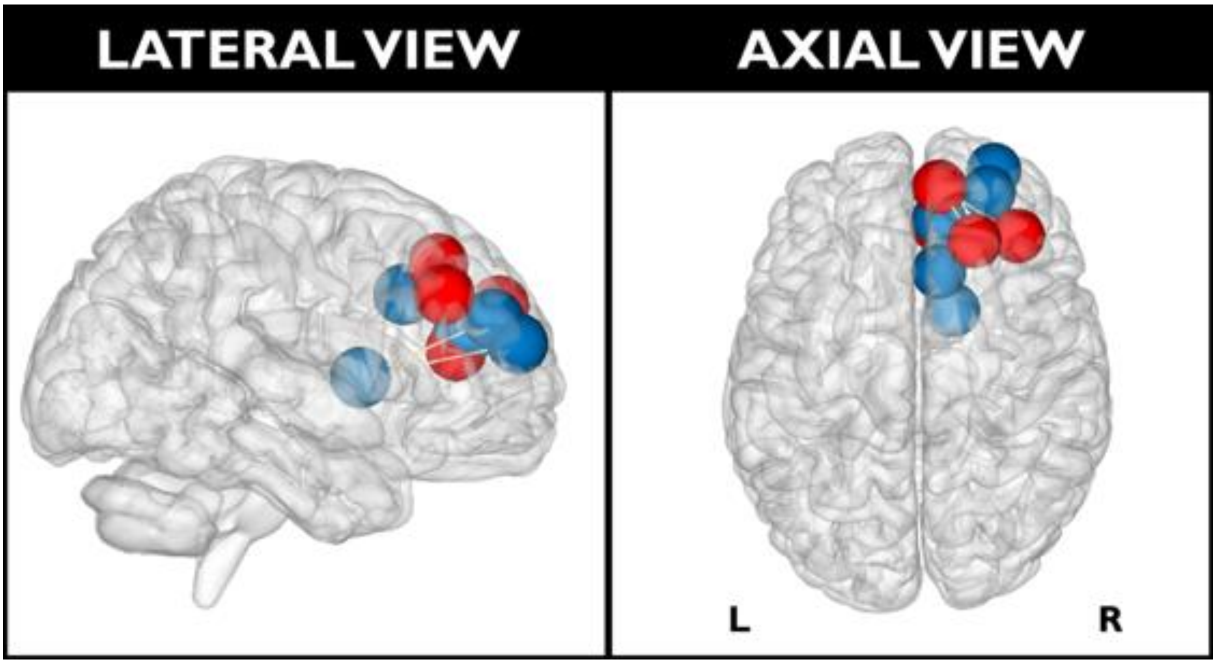

Figure S1. Graphical representation of the prefrontal-cingulum-striatal Dosenbach-meta-ROI and the prefrontal-cingular AAL-meta-ROI. The prefrontal-cingulum-striatal Dosenbach-meta-ROI is depicted in blue, while the AAL-based prefrontal-cingular meta-ROI is depicted in red.

Section 2.2: Results of the Spatial Characterization of Replicable Meta-ROI

The right prefrontal-cingulum-striatal meta-ROI showing a replicable association with cognitive measures overlapped with five NeuroMark components (R^2 > 0.02) involved in the cognitive-control network. These components encompassed the middle frontal gyrus (R^2 = 0.04 and R^2 = 0.03; respectively, components n.36 and n.41), middle cingulate cortex (R^2 = 0.03;

component n.39), superior medial frontal gyrus (R^2 = 0.02; component n.28) and the insula (R^2 = 0.02; component n.27) of the right hemisphere.

Section 2.3: Results of the Comparison Between Cohorts Consisted of Individuals with Subthreshold Psychotic Symptoms

Chi-square analyses on the distribution of psychopathological macrodomains in the different cohorts consisted of individuals with STPS show a similar distribution between the STPS cohort of the main investigation and PNC-STPS cohort in all the psychopathological macrodomains considered ($P > 0.05$), while the comparison between PRONIA-STPS and STPS cohort of the main investigation / PNC-STPS cohort shows a different distribution. In detail, the comparison between PRONIA-STPS and STPS of the main investigation shows a significantly different distribution of the macrodomain of psychosis (*Chi-squared* = 16.2 with Yates correction; $P < 0.001$) and depression (*Chi-squared* = 7.8 with Yates correction; $P = 0.005$). Comparison between PRONIA-STPS and PNC-STPS shows significantly different macrodomain distribution of psychosis (*Chi-squared* = 19.4 with Yates correction; $P < 0.001$), depression (*Chi-squared* = 9.2; $P = 0.002$) and mania (*Chi-squared* = 18.7 with Yates correction; $P < 0.001$). Sample sizes are reported in Table S3.

Section 2.4: Potential Sex-Related Differences in the Brain-Behaviour Association

In each study cohort, the results of Pearson's partial correlation analyses are reported in Table S4. These analyses aimed to investigate potential sex-related differences in the association between the degree centrality of the prefrontal-cingulate-striatal meta-ROI and executive performance.

The general linear models conducted in each study group of the main investigation revealed the main effect of meta-ROI degree centrality on executive performance. Specifically, NC discovery cohort showed a significant positive association between the degree centrality of the right prefrontal-cingulum-striatal meta-ROI and the WCST-TE ($T = 1.46$; *Wald Chi-squared* = 14.20; $P = 0.001$). The same brain-behavior association was significant in the NC replication cohort ($T = 0.79$; *Wald Chi-squared* = 6.72; $P = 0.005$). In the PSY cohort, the degree centrality of the right prefrontal-cingulum-striatal meta-ROI showed a marginally negative association with the WCST-TE scores ($T = -2.60$; *Wald Chi-squared* = 3.49; $P = 0.062$), while it was significantly negative in the STPS cohort ($T = -1.72$; *Wald Chi-squared* = 9.34; $P = 0.002$).

In the external replication phase, we found, in the PNC cohort, a positive association ($T = 0.01$; *Wald Chi-squared* = 8.60; $P = 0.002$) between the PCET-IR score and the degree centrality of

the prefrontal-cingulum-striatal meta-ROI in the PNC-TD group. Conversely, the same analysis in PNC-STPS indicated a negative association (T = -0.01; *Wald Chi-squared* = 3.02; P = 0.041). The association in the PNC-OD group was not significant (T = 0.01; *Wald Chi-squared* = 1.53; P = 0.216). Similarly, in the PRONIA cohort we found a positive association (T = 0.43; *Wald Chi-squared* = 3.07; P = 0.040) between the DSST-ICM and the degree centrality of the prefrontal-cingulum-striatal meta-ROI in the PRONIA-NC group. Conversely, we found again a negative association between DSST-ICM and degree centrality in the PRONIA-PSY group (T = -0.22; *Wald Chi-squared* = 4.63; P = 0.016). The association in the PRONIA-STPS group was not significant (T = -0.10; *Wald Chi-squared* = 0.66; P = 0.209). In the LIBD cohort we found a positive association (T = 0.17; *Wald Chi-squared* = 6.44; P = 0.006) between the WCST-TE and the degree centrality of the prefrontal-cingulum-striatal meta-ROI in the LIBD-NC group. Conversely, we found again a negative association between WCST-TE and degree centrality in the LIBD-PSY group (T = -0.80; *Wald Chi-squared* = 6.56; P = 0.005). The association in the LIBD-SIB group was not significant (T = 0.29; *Wald Chi-squared* = 0.33; P = 0.566). The interaction between the sex and the prefrontal-cingulum-striatal meta-ROI degree centrality on the cognitive performance was significant (*Wald Chi-squared* = 3.52; P = 0.031) only in the PNC-TD cohort. In the other cohorts of the study, the interaction was not significant ($P > 0.05$).

Section 2.5: Additional Brain-Behavior Associations and Differences Across Cohorts

The association between the degree centrality of the right prefrontal-cingulum-striatal meta-ROI and the NB-EI scores showed no significant correlations in the PSY and STPS cohorts (r = 0.26; P = 0.104 and r = 0.19; P = 0.427, respectively). The association between the degree centrality of the right prefrontal-cingulum-striatal meta-ROI and the WMS-TS scores showed no significant correlations in the PSY and STPS cohorts (r = 0.23; P = 0.157 and r = 0.14; P = 0.559, respectively). The association between the degree centrality of the right prefrontal-cingulum-striatal meta-ROI and the TMT-BA scores showed no significant correlations in the PSY and STPS cohorts (r = -0.01; P = 0.959 and r = -0.14; P = 0.563, respectively). The association between the degree centrality of the right prefrontal-cingulum-striatal meta-ROI and the CPT-FA scores showed no significant correlations in the PSY and STPS cohorts (r = -0.09; P = 0.562 and r = -0.27; P = 0.267, respectively).

The results of Pearson's partial correlation analyses, which examine the associations between the measures of centrality (betweenness centrality - degree centrality) of the 25 meta-ROIs and all neuropsychological scores in the two clinical cohorts of the main investigation are reported

in Table S2c-d. In the PSY cohort, we found a significant negative correlation (r = -0.59; pFDR = 0.007, Figure S2) between the degree centrality of a right ventromedial prefrontal meta-ROI (Figure 2 of the manuscript, right hemisphere: meta-ROI 2) and the total errors on Wisconsin Card Sorting Test. We tested this correlation in the clinical samples of the external replication datasets and found that it was not significantly replicable. Additionally, we found a positive correlation between the degree centrality of the same meta-ROI 2 and the total score of the Wechsler Memory Scale (r = 0.53; pFDR = 0.029). We replicated this correlation (r = 0.29; p = 0.031) in the PSY cohort of LIBD dataset. However, we could not estimate this correlation in the PNC and PRONIA datasets, as Wechsler Memory Scale data are not available in those samples. No statistically significant correlation was observed in the STPS sample.

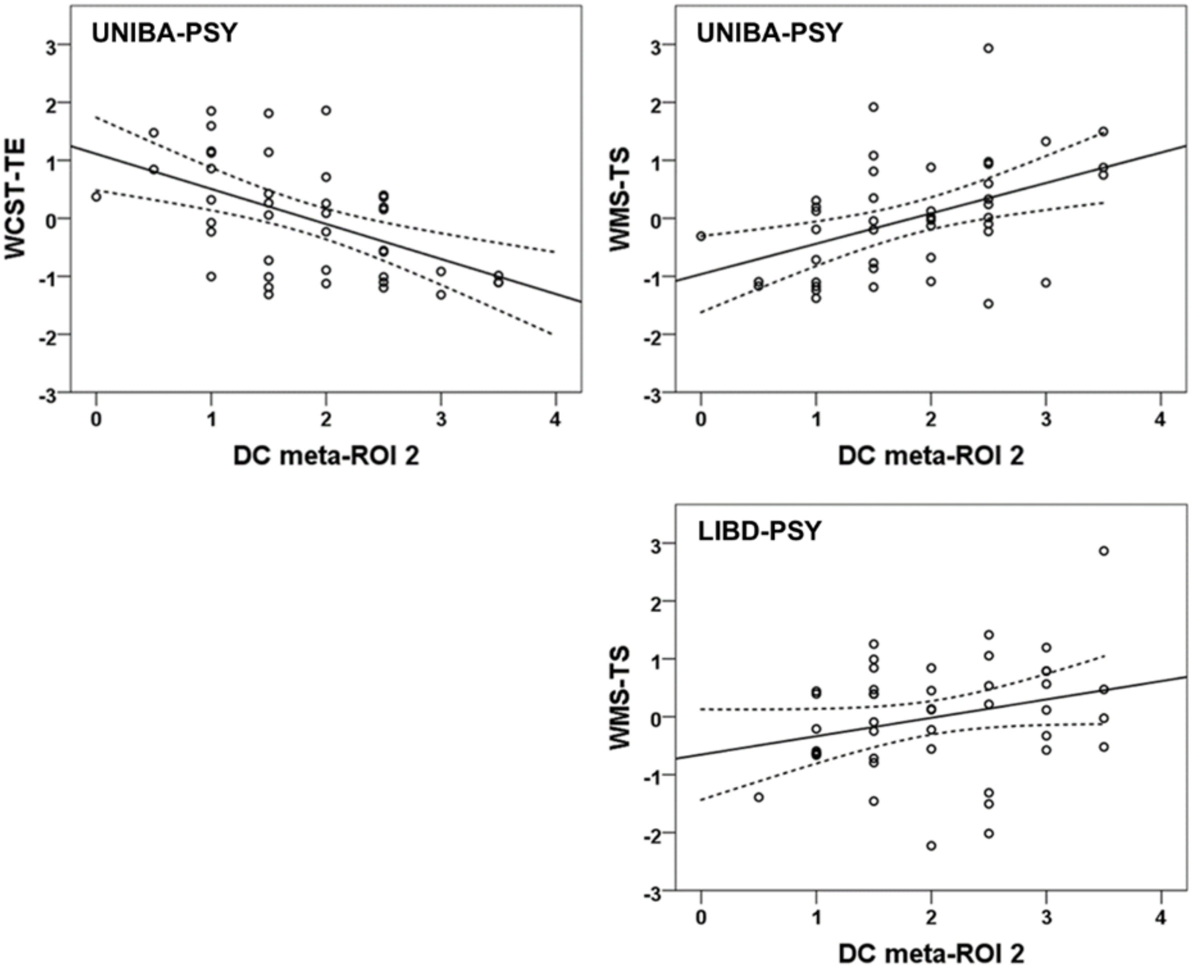

Figure S2. Association between the degree centrality of the ventromedial prefrontal meta-ROI and cognitive performance in the PSY cohort. The top panel of the figure shows the correlations estimated in the PSY cohort from the main investigation, while the bottom panel displays the correlation in the external replication dataset, LIBD. On the left, the association between meta-ROI degree centrality and the standardized residuals of WCST-TE is shown. On

the right, the associations between meta-ROI degree centrality and the standardized residuals of WMS-TS are depicted.

We have evaluated group-level differences in whole-brain centrality and in all neuropsychological variables (Table S3). Considering the betweenness centrality, we found differences among the three cohorts on a left cerebellum meta-ROI ($F(2,149) = 22.63$, pFDR = 0.001). Considering the degree centrality, we found differences among the three cohorts in the following meta-ROIs: left cerebellum meta-ROI ($F(2,149) = 16.01$, pFDR = 0.001), left precuneus-posterior cingulate meta-ROI ($F(2,149) = 7.66$, pFDR = 0.004), left parietal-occipital meta-ROI ($F(2,149) = 4.63$, pFDR = 0.040), left parietal-temporal-occipital meta-ROI ($F(2,149) = 6.77$, pFDR = 0.007), left frontal meta-ROI ($F(2,149) = 5.30$, pFDR = 0.025), right ventromedial prefrontal meta-ROI ($F(2,149) = 9.85$, pFDR = 0.001) and right cerebellum meta-ROI ($F(2,149) = 10.69$, pFDR = 0.001). We also tested these results in the external replication datasets. In the PRONIA dataset, we found differences in the right ventromedial prefrontal meta-ROI ($F(2,368) = 5.98$, pFDR = 0.035). Notably, this finding does not fully replicate the results of the UNIBA sample, given the dissimilar distribution of pairwise significant differences across groups, as evidenced by the post-hoc tests. No other differences were replicable in the external replication datasets. Bonferroni's post-hoc results are reported in the Table S3.

The ANOVA performed on the five neuropsychological scores across the three cohorts showed differences on all the neuropsychological scores, i.e., WCST-TE ($F(2,149) = 25.97$; pFDR = 0.001), NB-EI ($F(2,149) = 13.88$; pFDR = 0.001), WMS-TS ($F(2,149) = 17.40$; pFDR = 0.001), TMT-BA ($F(2,149) = 20.94$; pFDR = 0.001) and CPT-FA ($F(2,149) = 4.88$; pFDR = 0.001). We tested these results in the external replication datasets, finding that differences on the executive performance also emerged in the PRONIA (DSST-ICM: $F(2.368) = 58.23$, pFDR = 0.001) and LIBD (WCST-TE: $F(2,229) = 34.90$, pFDR = 0.001) samples. The results in the PRONIA sample do not fully replicate those of the UNIBA sample, as observed in the post-hoc tests. Conversely, post-hoc analyses conducted on the LIBD sample revealed a comparable pattern of differences with the UNIBA sample. However, it is important to keep in mind that this sample does not includes STPS subjects. Rather, siblings of patients are included. No differences were found in the PNC sample for PCET-IR. Also, we partially replicated between-group differences on the memory performance (WMS-TS: $F(2,139) = 31.07$; pFDR = 0.001) in the LIBD sample, as well as between-group differences on the working memory performance

(Auditory Digit Span – Correct Trials: $F(2,368) = 14.91$; $pFDR = 0.001$) in the PRONIA sample. Bonferroni-corrected post-hoc results are reported in the Table S3.

Supplementary Tables

Table S1: Imaging details of the cohorts included in this study.

Phase	Cohort	Scanner Type	sMRI				rs-fMRI				
			**SeqT**	**TR/TE (ms)**	**Vx (mm)**	**FOV (mm) /slice**	**SeqT**	**TR/TE (ms)**	**Vol**	**Vx (mm)**	**FOV (mm) /slice**
**Main investigation UNIBA**	MI-NCD	General Electric Signa 3T	MPRAGE	shortest	1x1x1.3	256/ 124	GE-EPI	2000/ 30	150	3.75x 3.75x5	240/26
	MI-NCR, MI-PSY, MI-STPS	Philips Ingenia 3T	T1-FFE	shortest	1x1x1	256/ 180	GE-EPI<br>GE-EPI	2000/ 30<br>3000/ 28	240<br>200	3x3x3.6<br>3x3x4	240/38<br>240/36
**External replication PNC**	PNC-TD, PNC-STPS, PNC-OD	Siemens 3T TIM Trio	MPRAGE	1810/ 3.5	0.9x0.9 x1	240/ 160	GE-EPI	3000/ 32	124	3x3x3	192/46
**External replication PRONIA**	PRONIA-NC, PRONIA-PSY, PRONIA-STPS	Philips Ingenia 3T: Munich and Cologne<br>Philips Achieva 3T: Cologne, Birmingham, Udine and Milan<br>Philips Ingenuity 3T: Turku<br>Siemens Prisma 3T and Siemens Verio: Basel	T1-FFE	shortest	1x1x1	256/ 180	GE-EPI	3000/ 28	200	3x3x4	240/36
**External replication LIBD**	LIBD-NC, LIBD-PSY, LIBD-SIB	General Electric Signa 3T	MPRAGE	shortest	1x1x1.3	256/ 124	GE-EPI	2000/ 30	300	4x4x5	240/26

Abbreviations: MI: Main investigation of the study using UNIBA dataset; MI-NCD: Neurotypical controls in the discovery cohort of the main investigation; MI-NCR: Neurotypical controls in within-site replication cohort of the main investigation; MI-PSY: Chronic patients with psychosis of the main investigation; MI-STPS: Individuals with subthreshold psychotic symptoms of the main investigation; PNC-TD: Individuals with typical development in PNC dataset; PNC-STPS: Individuals with a developmental trajectory towards psychotic disorders in PNC dataset; PNC-OD: Individuals with a developmental trajectory towards other psychiatric disorders in PNC dataset; PRONIA-NC: Neurotypical controls in PRONIA dataset; PRONIA-PSY: Patients with a recent-onset psychosis in PRONIA dataset; PRONIA-STPS: Individuals with subthreshold psychotic symptoms in PRONIA dataset; LIBD-NC: Neurotypical controls in LIBD dataset; LIBD-PSY: Patients with schizophrenia in LIBD dataset; LIBD-SIB: Unaffected siblings of patients with schizophrenia in LIBD dataset; sMRI: Structural magnetic resonance imaging; rs-fMRI: Resting state - functional magnetic resonance imaging; SeqT: MRI sequence type; TR: Repetition time; TE: Echo time; Vx: Voxel size; FOV: Field of view; Vol: number of volumes.

Table S2: Pearson's partial correlations between meta-ROI betweenness centrality / degree centrality values and neuropsychological scores in the main investigation cohorts.

2a. Pearson's partial correlations in MI-NCD (Cov: Age and sex; *DoF* = 113)

META-ROI	WCST-TE				NB-EI				WMS-TS				TMT-BA				CPT-FA			
	BC		DC		BC		DC		BC		DC		BC		DC		BC		DC	
	r	P	r	P	r	P	r	P	r	P	r	P	r	P	r	P	r	P	r	P
**1 (LH)**	-0.03	0.74	-0.10	0.31	-0.04	0.71	-0.01	0.90	0.09	0.37	-0.13	0.19	0.12	0.21	-0.01	0.92	0.10	0.29	0.03	0.75
**2 (LH)**	-0.07	0.47	-0.08	0.39	-0.07	0.43	-0.13	0.18	0.06	0.50	0.10	0.28	-0.13	0.16	-0.01	0.89	0.07	0.47	0.04	0.70
**3 (LH)**	0.15	0.11	0.14	0.13	-0.02	0.80	0.05	0.61	0.01	0.92	0.02	0.87	-0.01	0.92	0.02	0.85	-0.02	0.80	0.14	0.13
**4 (LH)**	0.01	0.95	0.09	0.33	-0.07	0.49	-0.10	0.29	0.03	0.72	-0.01	0.99	0.01	0.97	0.01	0.91	-0.05	0.62	0.02	0.85
**5 (LH)**	0.08	0.42	-0.01	0.92	-0.02	0.80	0.20	0.03	-0.01	0.99	0.24	0.01	0.02	0.84	-0.09	0.32	-0.03	0.79	0.12	0.21
**6 (LH)**	-0.14	0.14	0.02	0.82	0.13	0.18	0.21	0.03	-0.12	0.20	0.15	0.11	0.07	0.47	-0.02	0.80	-0.03	0.78	0.12	0.20
**7 (LH)**	-0.09	0.36	0.13	0.17	0.07	0.48	0.07	0.49	0.11	0.23	-0.04	0.70	0.01	0.93	0.03	0.74	0.14	0.15	0.07	0.48
**8 (LH)**	-0.06	0.51	-0.11	0.23	0.01	0.91	-0.13	0.18	0.18	0.05	0.18	0.06	-0.01	0.90	-0.01	0.89	0.07	0.44	-0.02	0.81
**9 (LH)**	-0.12	0.22	0.10	0.31	0.04	0.66	0.01	0.90	-0.09	0.32	0.04	0.68	-0.10	0.30	0.02	0.84	0.02	0.82	-0.13	0.18
**10 (LH)**	-0.05	0.60	0.17	0.08	0.11	0.25	-0.04	0.67	0.05	0.61	0.03	0.75	-0.19	0.05	-0.02	0.80	-0.04	0.66	-0.12	0.21
**11 (LH)**	0.10	0.28	0.15	0.12	-0.07	0.46	-0.08	0.39	0.09	0.32	-0.09	0.36	-0.09	0.35	0.13	0.18	-0.01	0.91	0.06	0.51
**12 (LH)**	-0.06	0.54	0.08	0.42	-0.08	0.39	-0.14	0.15	-0.04	0.70	-0.01	0.98	0.03	0.73	0.08	0.43	0.12	0.21	0.06	0.52
**1 (RH)**	-0.21	0.03	0.03	0.79	-0.07	0.48	0.03	0.74	0.11	0.24	0.07	0.44	0.04	0.71	-0.03	0.72	0.08	0.42	-0.01	0.89
**2 (RH)**	0.01	0.94	0.16	0.10	0.11	0.24	0.10	0.31	-0.01	0.95	0.07	0.45	-0.08	0.38	0.06	0.56	0.09	0.35	0.08	0.39
**3 (RH)**	0.01	0.30	**0.33**	**0.01**	-0.07	0.44	-0.06	0.51	0.10	0.30	0.01	0.90	0.01	0.99	0.09	0.35	-0.03	0.76	0.01	0.92
**4 (RH)**	-0.01	0.88	0.03	0.75	0.08	0.38	-0.05	0.60	0.05	0.57	-0.05	0.57	0.06	0.51	0.11	0.23	-0.11	0.23	-0.03	0.73
**5 (RH)**	-0.04	0.68	0.05	0.61	-0.03	0.72	0.11	0.25	-0.10	0.28	-0.11	0.26	0.13	0.19	-0.03	0.76	-0.14	0.14	-0.09	0.34
**6 (RH)**	0.05	0.57	-0.03	0.75	0.01	0.89	0.10	0.30	0.13	0.16	0.08	0.40	-0.01	0.96	-0.12	0.19	-0.10	0.28	-0.04	0.66
**7 (RH)**	-0.09	0.35	-0.05	0.61	0.01	0.10	0.09	0.33	0.14	0.14	0.14	0.14	-0.04	0.64	0.01	0.88	0.01	0.89	0.03	0.77
**8 (RH)**	0.01	0.97	0.15	0.12	0.04	0.70	0.23	0.01	-0.13	0.18	0.05	0.60	0.10	0.28	0.02	0.87	-0.12	0.19	0.01	0.97
**9 (RH)**	-0.08	0.38	0.05	0.63	-0.10	0.27	0.09	0.35	0.01	0.89	-0.04	0.69	-0.21	0.02	-0.03	0.74	-0.03	0.74	0.05	0.63
**10(RH)**	-0.10	0.29	0.19	0.05	-0.08	0.39	0.06	0.52	0.05	0.57	-0.03	0.72	-0.03	0.75	0.14	0.13	-0.10	0.27	-0.02	0.85
**11(RH)**	0.01	0.98	-0.10	0.29	-0.04	0.66	-0.05	0.63	-0.10	0.27	-0.14	0.14	0.04	0.65	-0.02	0.80	0.13	0.16	-0.01	0.91
**12(RH)**	0.11	0.27	0.04	0.71	0.01	0.97	-0.07	0.44	0.05	0.62	-0.10	0.31	0.06	0.50	0.04	0.69	0.10	0.30	0.03	0.73
**13(RH)**	0.03	0.76	-0.07	0.44	0.08	0.42	0.06	0.54	0.03	0.74	0.06	0.55	-0.10	0.28	0.07	0.47	0.01	0.88	-0.13	0.16

2b. Pearson's partial correlations in MI-NCR (Cov: Age, sex and TR; *DoF* = 82)

**META-ROI**	**WCST-TE**				**NB-EI**				**WMS-TS**				**TMT-BA**				**CPT-FA**			
	**BC**		**DC**		**BC**		**DC**		**BC**		**DC**		**BC**		**DC**		**BC**		**DC**	
	**r**	**P**	**r**	**P**	**r**	**P**	**r**	**P**	**r**	**P**	**r**	**P**	**r**	**P**	**r**	**P**	**r**	**P**	**r**	**P**
**1 (LH)**	-0.15	0.15	0.23	0.02	0.15	0.09	-0.11	0.15	0.08	0.25	-0.06	0.29	-0.03	0.38	-0.03	0.38	0.12	0.15	0.01	0.48
**2 (LH)**	0.11	0.16	0.03	0.41	-0.04	0.36	0.08	0.24	0.06	0.28	-0.14	0.10	-0.02	0.42	0.08	0.23	0.06	0.30	0.18	0.05
**3 (LH)**	0.14	0.10	0.15	0.09	0.04	0.37	-0.21	0.03	-0.04	0.35	0.01	0.50	-0.00	0.49	0.11	0.16	0.19	0.04	0.23	0.02
**4 (LH)**	0.05	0.33	0.26	0.01	-0.08	0.24	0.05	0.34	0.02	0.42	-0.14	0.11	0.23	0.02	-0.20	0.03	0.03	0.39	0.17	0.06
**5 (LH)**	-0.12	0.14	0.26	0.01	-0.07	0.27	-0.11	0.16	0.02	0.43	0.02	0.42	-0.02	0.44	0.08	0.24	-0.10	0.19	0.14	0.10
**6 (LH)**	0.03	0.39	0.28	0.01	0.10	0.19	-0.08	0.25	-0.24	0.01	-0.28	0.01	0.01	0.49	0.14	0.10	-0.07	0.27	0.13	0.12
**7 (LH)**	-0.15	0.09	0.03	0.40	-0.07	0.26	0.03	0.39	0.04	0.38	0.09	0.22	-0.02	0.42	-0.08	0.24	-0.26	0.01	0.18	0.05
**8 (LH)**	0.09	0.21	0.08	0.25	-0.12	0.14	0.10	0.19	-0.10	0.18	-0.11	0.16	0.14	0.10	0.01	0.48	0.05	0.34	0.02	0.43
**9 (LH)**	0.07	0.28	0.25	0.01	0.05	0.34	0.10	0.18	-0.00	0.49	0.01	0.50	0.01	0.50	0.01	0.48	-0.08	0.23	0.21	0.03
**10 (LH)**	-0.08	0.23	0.22	0.02	-0.02	0.43	0.07	0.26	-0.08	0.24	-0.02	0.45	-0.01	0.47	-0.12	0.14	-0.01	0.46	0.23	0.02
**11 (LH)**	-0.07	0.25	0.16	0.07	0.03	0.41	0.07	0.26	0.18	0.05	-0.06	0.30	-0.05	0.34	-0.17	0.06	-0.10	0.18	0.18	0.05
**12 (LH)**	-0.01	0.45	0.23	0.02	-0.09	0.22	-0.13	0.18	0.02	0.43	0.05	0.33	-0.15	0.08	-0.13	0.12	-0.02	0.44	0.19	0.04
**1 (RH)**	-0.09	0.21	-0.09	0.21	0.32	0.01	0.01	0.49	-0.08	0.23	0.16	0.07	-0.08	0.22	-0.04	0.38	-0.06	0.31	0.09	0.21
**2 (RH)**	-0.03	0.39	0.10	0.18	-0.11	0.16	0.22	0.03	-0.07	0.25	-0.07	0.26	0.01	0.48	0.07	0.26	-0.02	0.42	0.28	0.01
**3 (RH)**	0.30	0.00	0.27	0.01	0.04	0.34	0.09	0.20	-0.06	0.29	0.12	0.15	-0.11	0.16	-0.19	0.04	-0.09	0.22	0.11	0.17
**4 (RH)**	0.10	0.19	-0.19	0.04	-0.03	0.40	0.16	0.08	-0.01	0.46	0.06	0.30	0.04	0.37	0.05	0.32	0.15	0.08	-0.16	0.08
**5 (RH)**	0.06	0.30	-0.19	0.04	0.06	0.28	-0.05	0.34	0.05	0.34	0.03	0.38	0.03	0.40	0.15	0.09	0.13	0.13	0.05	0.34
**6 (RH)**	-0.09	0.20	-0.34	0.01	0.11	0.17	0.07	0.28	0.06	0.30	0.01	0.47	-0.06	0.30	0.08	0.25	-0.01	0.49	-0.19	0.04
**7 (RH)**	0.01	0.46	-0.25	0.01	-0.21	0.03	0.04	0.36	-0.09	0.20	0.04	0.34	0.05	0.34	0.12	0.14	0.01	0.47	-0.23	0.02
**8 (RH)**	0.02	0.43	-0.13	0.12	0.23	0.02	-0.13	0.13	-0.05	0.34	0.20	0.04	0.02	0.42	0.17	0.06	-0.01	0.47	0.01	0.50
**9 (RH)**	-0.03	0.41	0.01	0.48	0.10	0.18	-0.15	0.08	-0.02	0.42	0.07	0.26	0.08	0.23	0.19	0.04	-0.11	0.17	0.06	0.29
**10(RH)**	0.01	0.45	-0.18	0.05	-0.04	0.35	-0.10	0.17	-0.09	0.21	-0.01	0.48	0.17	0.07	0.11	0.16	0.07	0.26	-0.09	0.22
**11(RH)**	-0.01	0.50	0.01	0.49	-0.06	0.30	-0.11	0.17	0.01	0.46	0.01	0.49	0.01	0.46	0.08	0.23	0.15	0.09	-0.18	0.05
**12(RH)**	0.19	0.04	-0.09	0.20	-0.04	0.38	-0.01	0.47	0.02	0.43	0.05	0.31	-0.02	0.43	-0.09	0.21	-0.01	0.46	-0.18	0.06
**13(RH)**	0.01	0.48	-0.26	0.01	-0.10	0.18	0.06	0.28	-0.16	0.08	0.07	0.26	0.05	0.34	-0.01	0.50	0.07	0.27	-0.11	0.15

2c. Pearson's partial correlations in MI-PSY (Cov: Age, sex and TR; DoF = 38)																				
**META-ROI**	**WCST-TE**				**NB-EI**				**WMS-TS**				**TMT-BA**				**CPT-FA**			
	**BC**		**DC**		**BC**		**DC**		**BC**		**DC**		**BC**		**DC**		**BC**		**DC**	
	**r**	**P**	**r**	**P**	**r**	**P**	**r**	**P**	**r**	**P**	**r**	**P**	**r**	**P**	**r**	**P**	**r**	**P**	**r**	**P**
**1 (LH)**	-0.01	0.97	0.08	0.62	0.06	0.74	0.20	0.20	0.09	0.57	-0.22	0.17	-0.13	0.42	0.21	0.20	0.02	0.90	0.13	0.43
**2 (LH)**	-0.35	0.02	-0.01	0.99	-0.05	0.78	-0.16	0.32	0.21	0.19	-0.02	0.93	-0.10	0.55	0.02	0.89	-0.18	0.27	-0.16	0.32
**3 (LH)**	-0.01	0.96	-0.04	0.81	-0.11	0.50	0.32	0.04	0.02	0.88	0.18	0.27	-0.11	0.48	-0.13	0.43	0.05	0.77	-0.03	0.85
**4 (LH)**	-0.03	0.86	-0.14	0.39	0.13	0.44	0.20	0.23	0.07	0.65	0.05	0.77	0.04	0.81	0.02	0.92	-0.03	0.87	0.03	0.87
**5 (LH)**	-0.12	0.47	-0.19	0.23	0.12	0.46	0.08	0.64	0.21	0.19	0.17	0.29	-0.27	0.09	-0.01	0.97	0.45	0.01	-0.16	0.34
**6 (LH)**	-0.02	0.92	0.05	0.77	-0.01	0.94	-0.01	0.99	0.18	0.28	-0.15	0.35	-0.07	0.65	0.12	0.47	-0.07	0.66	-0.10	0.52
**7 (LH)**	-0.13	0.42	-0.21	0.20	-0.03	0.84	0.05	0.77	0.12	0.46	0.20	0.21	-0.12	0.45	-0.05	0.77	0.23	0.16	0.04	0.81
**8 (LH)**	0.11	0.50	-0.02	0.89	0.01	0.93	-0.19	0.24	-0.01	0.96	0.03	0.85	0.20	0.22	0.11	0.48	0.24	0.14	0.09	0.57
**9 (LH)**	0.16	0.32	-0.11	0.48	-0.02	0.93	0.11	0.50	0.03	0.85	0.27	0.10	0.01	0.97	-0.08	0.61	0.09	0.56	0.03	0.83
**10 (LH)**	-0.12	0.45	-0.18	0.27	0.02	0.90	0.13	0.42	0.20	0.21	0.25	0.12	0.02	0.92	-0.06	0.73	0.08	0.63	0.15	0.34
**11 (LH)**	-0.09	0.59	-0.16	0.33	0.07	0.65	0.12	0.45	0.38	0.02	0.24	0.14	-0.07	0.69	-0.10	0.55	-0.19	0.25	-0.02	0.89
**12 (LH)**	0.04	0.80	-0.17	0.30	0.10	0.54	0.11	0.49	0.35	0.03	0.19	0.25	-0.20	0.22	0.15	0.35	-0.06	0.71	-0.10	0.54
**1 (RH)**	0.08	0.62	-0.16	0.32	-0.08	0.64	-0.09	0.57	-0.05	0.74	0.11	0.48	0.04	0.82	0.10	0.55	0.17	0.29	0.04	0.82
**2 (RH)**	-0.21	0.19	**-0.59**	**0.01**	0.08	0.63	0.37	0.02	0.07	0.66	**0.53***	**0.01**	-0.12	0.46	-0.35	0.02	-0.01	0.98	-0.20	0.21
**3 (RH)**	-0.16	0.32	-0.37	0.02	0.30	0.06	0.26	0.10	0.22	0.17	0.23	0.16	-0.05	0.75	-0.01	0.96	-0.10	0.53	-0.09	0.56
**4 (RH)**	-0.04	0.80	0.14	0.38	-0.10	0.54	0.06	0.72	0.01	0.97	-0.09	0.60	0.25	0.13	0.09	0.59	-0.15	0.35	0.08	0.63
**5 (RH)**	0.11	0.51	0.15	0.35	-0.09	0.58	0.13	0.44	-0.04	0.82	-0.08	0.62	0.08	0.61	-0.13	0.44	-0.15	0.36	-0.07	0.66
**6 (RH)**	0.03	0.85	0.17	0.30	-0.04	0.79	-0.14	0.39	0.06	0.72	-0.03	0.87	0.20	0.21	-0.13	0.41	0.15	0.37	-0.01	0.98
**7 (RH)**	-0.01	0.94	0.22	0.17	-0.04	0.79	-0.20	0.23	-0.19	0.25	-0.27	0.10	0.28	0.08	-0.04	0.79	-0.08	0.61	0.03	0.86
**8 (RH)**	0.18	0.28	0.12	0.45	-0.15	0.36	-0.35	0.02	-0.20	0.23	-0.29	0.07	0.09	0.58	0.18	0.26	0.01	0.96	0.01	0.94
**9 (RH)**	0.06	0.70	0.01	0.98	-0.15	0.36	0.31	0.05	-0.12	0.47	0.10	0.53	0.18	0.27	-0.05	0.76	-0.17	0.28	-0.11	0.49
**10(RH)**	0.13	0.43	0.08	0.64	0.11	0.49	-0.07	0.68	-0.08	0.63	-0.18	0.26	0.05	0.75	0.07	0.69	-0.01	0.97	0.12	0.46
**11(RH)**	-0.22	0.17	0.13	0.43	0.29	0.07	0.07	0.69	0.18	0.26	-0.17	0.30	-0.06	0.69	-0.05	0.76	-0.14	0.39	0.04	0.81
**12(RH)**	-0.01	0.98	0.21	0.19	-0.06	0.72	-0.20	0.22	-0.15	0.36	-0.22	0.18	0.08	0.62	0.07	0.65	0.02	0.90	-0.11	0.51
**13(RH)**	-0.36	0.02	-0.07	0.66	0.27	0.09	-0.07	0.66	0.12	0.47	0.12	0.46	-0.21	0.19	-0.17	0.28	-0.23	0.16	-0.11	0.48

*We replicated this correlation (r = 0.29; p = 0.031) in the PSY cohort of LIBD dataset.

2d. Pearson's partial correlations in MI-STPS (Cov: Age, sex and TR; DoF = 17)																				
**META-ROI**	**WCST-TE**				**NB-EI**				**WMS-TS**				**TMT-BA**				**CPT-FA**			
	**BC**		**DC**		**BC**		**DC**		**BC**		**DC**		**BC**		**DC**		**BC**		**DC**	
	**r**	**P**	**r**	**P**	**r**	**P**	**r**	**P**	**r**	**P**	**r**	**P**	**r**	**P**	**r**	**P**	**r**	**P**	**r**	**P**
**1 (LH)**	-0.36	0.13	0.28	0.24	-0.07	0.77	-0.12	0.64	-0.34	0.15	0.09	0.73	-0.29	0.23	0.07	0.78	-0.44	0.06	0.13	0.61
**2 (LH)**	-0.21	0.39	-0.01	0.97	-0.26	0.28	0.01	0.97	-0.06	0.82	-0.15	0.54	0.01	0.99	0.30	0.21	-0.16	0.52	0.31	0.20
**3 (LH)**	0.03	0.91	0.20	0.41	0.18	0.46	-0.11	0.65	0.18	0.47	-0.14	0.58	0.40	0.09	-0.03	0.90	0.22	0.38	0.02	0.93
**4 (LH)**	0.38	0.11	0.31	0.20	-0.10	0.69	-0.33	0.17	0.08	0.73	0.11	0.65	0.66	0.01	0.24	0.32	0.28	0.25	-0.06	0.79
**5 (LH)**	-0.09	0.71	-0.35	0.15	-0.01	0.95	0.13	0.61	0.50	0.03	0.17	0.48	0.15	0.54	0.16	0.52	0.15	0.53	-0.11	0.65
**6 (LH)**	-0.07	0.79	-0.08	0.74	-0.40	0.09	-0.20	0.42	-0.03	0.90	-0.02	0.94	-0.34	0.15	-0.23	0.35	0.05	0.83	-0.32	0.18
**7 (LH)**	-0.45	0.05	-0.27	0.26	0.18	0.47	0.23	0.35	0.48	0.04	-0.21	0.39	0.11	0.66	0.02	0.94	-0.12	0.62	-0.16	0.51
**8 (LH)**	0.24	0.31	0.16	0.50	-0.13	0.60	-0.04	0.88	0.02	0.93	-0.21	0.39	0.68	0.01	0.05	0.84	0.23	0.34	-0.06	0.79
**9 (LH)**	-0.09	0.71	-0.29	0.23	-0.25	0.31	0.20	0.41	-0.32	0.17	-0.05	0.85	0.01	0.98	-0.23	0.34	0.25	0.31	-0.04	0.89
**10 (LH)**	0.01	0.99	0.07	0.77	0.09	0.71	0.08	0.75	-0.31	0.19	0.09	0.70	-0.26	0.29	-0.18	0.46	0.08	0.73	-0.04	0.87
**11 (LH)**	0.37	0.12	0.45	0.05	-0.11	0.65	-0.11	0.64	0.09	0.72	-0.08	0.73	0.39	0.10	0.07	0.77	0.44	0.06	-0.01	0.99
**12 (LH)**	0.12	0.62	-0.21	0.40	0.42	0.07	0.20	0.41	0.27	0.26	0.24	0.32	-0.29	0.23	-0.34	0.16	0.34	0.16	-0.26	0.28
**1 (RH)**	-0.18	0.45	-0.25	0.30	-0.09	0.70	-0.24	0.32	0.15	0.53	0.01	0.99	0.16	0.52	0.11	0.66	0.12	0.62	0.08	0.75
**2 (RH)**	0.07	0.77	-0.17	0.49	-0.21	0.39	0.33	0.17	0.06	0.81	0.39	0.10	0.48	0.04	0.01	0.97	0.16	0.51	0.13	0.61
**3 (RH)**	-0.01	0.98	-0.59	0.01	0.14	0.57	0.19	0.43	0.23	0.33	0.14	0.56	0.57	0.01	-0.14	0.56	0.01	0.96	-0.27	0.27
**4 (RH)**	0.09	0.71	-0.04	0.86	-0.06	0.80	-0.22	0.37	0.11	0.66	-0.16	0.51	0.07	0.77	0.05	0.82	0.17	0.48	-0.42	0.07
**5 (RH)**	0.30	0.21	-0.03	0.92	-0.05	0.84	-0.12	0.63	-0.01	0.98	-0.13	0.60	0.22	0.36	0.13	0.60	0.26	0.28	0.04	0.87
**6 (RH)**	-0.04	0.86	-0.09	0.70	0.01	0.97	-0.23	0.34	0.54	0.02	0.03	0.89	-0.08	0.74	-0.06	0.80	0.35	0.14	0.22	0.36
**7 (RH)**	0.05	0.83	-0.15	0.54	0.22	0.37	-0.07	0.77	-0.35	0.15	0.04	0.88	0.22	0.36	0.07	0.78	-0.19	0.45	-0.24	0.33
**8 (RH)**	-0.02	0.94	-0.25	0.31	0.09	0.70	0.23	0.33	-0.21	0.40	-0.26	0.28	-0.27	0.27	-0.05	0.84	-0.28	0.25	-0.33	0.17
**9 (RH)**	0.10	0.69	0.03	0.91	-0.13	0.61	-0.25	0.30	0.04	0.87	0.01	0.98	0.22	0.37	-0.01	0.97	0.34	0.16	-0.07	0.77
**10(RH)**	0.22	0.36	-0.25	0.31	0.01	0.98	-0.18	0.46	0.21	0.38	0.32	0.19	0.08	0.74	0.12	0.62	0.28	0.24	-0.10	0.68
**11(RH)**	-0.33	0.17	0.37	0.12	-0.29	0.23	0.16	0.52	-0.40	0.09	0.36	0.13	-0.29	0.23	0.27	0.26	-0.40	0.09	0.34	0.15
**12(RH)**	0.06	0.81	-0.01	0.98	-0.15	0.54	0.09	0.72	0.14	0.55	-0.10	0.68	-0.08	0.75	-0.30	0.22	-0.07	0.78	-0.05	0.84
**13(RH)**	-0.28	0.24	-0.14	0.57	-0.24	0.31	0.33	0.17	-0.20	0.42	-0.04	0.86	-0.05	0.84	-0.19	0.44	-0.22	0.36	0.02	0.92

Abbreviations: MI-NCD: Neurotypical controls in discovery cohort of the main investigation study; MI-NCR: Neurotypical controls in within-site replication cohort of the main investigation study; MI-PSY: Chronic patients with psychosis of the main investigation study; MI-STPS: Individuals with subthreshold psychotic symptoms of the main investigation study; BC: Betweenness centrality; DC: Degree centrality; DoF: Degree of freedom; Cov: Covariates; WCST-TE: Number of total errors at Wisconsin Card Sorting Test; NB-EI: N-Back efficiency index; WMS-TS: Wechsler Memory Scale - Total score; TMT-BA: Trail Making Test - difference between Part B and part A; CPT-FA: Continuous Performance Test - False alarms ratio; LH: Left hemisphere; RH: right hemisphere. Correlations that encompass the threshold of *pFDR* < 0.05 are shown in bold type.

Table S3: Group-level differences in whole-brain centrality and in all neuropsychological variables. ANOVAs were employed to evaluate the differences (pFDR < 0.05) in centrality measures and neuropsychological scores. The FDR correction was applied separately for betweenness centrality, degree centrality and neuropsychological analyses. Bonferroni's post-hoc analyses (P < 0.05) were employed to observe pairwise differences.

MAIN INVESTIGATION: UNIBA									
**VARIABLES**	**ANOVA**			**MI-NCR vs MI-PSY**		**MI-NCR vs MI-STPS**		**MI-PSY vs MI-STPS**	
	**F (2, 149)**	**P**	**pFDR**	**t(128)**	**P**	**t(107)**	**P**	**t(63)**	**P**
BC META-ROI 1 (LH)	22.63	0.01	0.01	-5.24	0.01	-5.53	0.01	-1.44	0.40
BC META-ROI 2 (LH)	1.64	0.20	0.55	-	-	-	-	-	-
BC META-ROI 3 (LH)	2.38	0.10	0.40	-	-	-	-	-	-
BC META-ROI 4 (LH)	1.70	0.19	0.55	-	-	-	-	-	-
BC META-ROI 5 (LH)	0.17	0.84	0.88	-	-	-	-	-	-
BC META-ROI 6 (LH)	1.47	0.23	0.57	-	-	-	-	-	-
BC META-ROI 7 (LH)	0.31	0.74	0.88	-	-	-	-	-	-
BC META-ROI 8 (LH)	1.98	0.14	0.51	-	-	-	-	-	-
BC META-ROI 9 (LH)	0.45	0.64	0.88	-	-	-	-	-	-
BC META-ROI 10 (LH)	0.59	0.56	0.88	-	-	-	-	-	-
BC META-ROI 11 (LH)	0.50	0.61	0.88	-	-	-	-	-	-
BC META-ROI 12 (LH)	0.57	0.57	0.88	-	-	-	-	-	-
BC META-ROI 1 (RH)	0.37	0.69	0.88	-	-	-	-	-	-
BC META-ROI 2 (RH)	0.33	0.72	0.88	-	-	-	-	-	-
BC META-ROI 3 (RH)	3.94	0.02	0.11	-	-	-	-	-	-
BC META-ROI 4 (RH)	1.11	0.33	0.69	-	-	-	-	-	-
BC META-ROI 5 (RH)	0.17	0.84	0.88	-	-	-	-	-	-
BC META-ROI 6 (RH)	0.23	0.79	0.88	-	-	-	-	-	-
BC META-ROI 7 (RH)	0.38	0.69	0.88	-	-	-	-	-	-
BC META-ROI 8 (RH)	0.17	0.84	0.88	-	-	-	-	-	-
BC META-ROI 9 (RH)	1.39	0.25	0.57	-	-	-	-	-	-
BC META-ROI 10 (RH)	0.02	0.98	0.98	-	-	-	-	-	-
BC META-ROI 11 (RH)	5.06	0.01	0.08	-	-	-	-	-	-

BC META-ROI 12 (RH)	4.83	0.01	0.08	-	-	-	-	-	-
BC META-ROI 13 (RH)	4.36	0.01	0.09	-	-	-	-	-	-
DC META-ROI 1 (LH)	16.01	0.01	0.01	-5.14	0.01	-3.31	0.01	0.55	0.99
DC META-ROI 2 (LH)	1.72	0.18	0.29	-	-	-	-	-	-
DC META-ROI 3 (LH)	7.66	0.01	0.01	3.06	0.01	3.18	0.01	0.67	0.99
DC META-ROI 4 (LH)	0.70	0.50	0.59	-	-	-	-	-	-
DC META-ROI 5 (LH)	2.63	0.08	0.21	-	-	-	-	-	-
DC META-ROI 6 (LH)	4.63	0.01	0.04	2.00	0.13	2.61	0.02	1.14	0.93
DC META-ROI 7 (LH)	0.59	0.56	0.63	-	-	-	-	-	-
DC META-ROI 8 (LH)	6.77	0.01	0.01	3.56	0.01	-0.09	0.99	-2.76	0.03
DC META-ROI 9 (LH)	5.30	0.01	0.02	2.98	0.01	2.13	0.10	-0.10	0.99
DC META-ROI 10 (LH)	2.37	0.10	0.21	-	-	-	-	-	-
DC META-ROI 11 (LH)	1.19	0.31	0.42	-	-	-	-	-	-
DC META-ROI 12 (LH)	0.30	0.74	0.74	-	-	-	-	-	-
DC META-ROI 1 (RH)	2.01	0.14	0.23	-	-	-	-	-	-
DC META-ROI 2 (RH)	9.85	0.01	0.01	3.55	0.01	3.39	0.01	0.75	0.99
DC META-ROI 3 (RH)	1.54	0.22	0.32	-	-	-	-	-	-
DC META-ROI 4 (RH)	0.35	0.71	0.74	-	-	-	-	-	-
DC META-ROI 5 (RH)	1.14	0.32	0.42	-	-	-	-	-	-
DC META-ROI 6 (RH)	0.33	0.72	0.74	-	-	-	-	-	-
DC META-ROI 7 (RH)	2.09	0.13	0.23	-	-	-	-	-	-
DC META-ROI 8 (RH)	2.32	0.10	0.21	-	-	-	-	-	-
DC META-ROI 9 (RH)	3.60	0.03	0.09	-	-	-	-	-	-
DC META-ROI 10 (RH)	2.48	0.09	0.21	-	-	-	-	-	-
DC META-ROI 11 (RH)	10.69	0.01	0.01	-4.41	0.01	-2.22	0.05	1.17	0.98
DC META-ROI 12 (RH)	2.24	0.11	0.21	-	-	-	-	-	-
DC META-ROI 13 (RH)	1.01	0.37	0.46	-	-	-	-	-	-
WCST-TE	25.97	0.01	0.01	-7.20	0.01	-2.86	0.15	2.42	0.01
NB-EI	13.88	0.01	0.01	4.87	0.01	2.44	0.03	-1.69	0.69
WMS-TS	17.40	0.01	0.01	6.01	0.01	3.44	0.01	-0.83	0.96

TMT-BA	20.94	0.01	0.01	-6.42	0.01	-5.32	0.01	-0.24	0.99
CPT-FA	4.88	0.01	0.01	-2.79	0.02	-3.14	0.11	0.03	0.99
**EXTERNAL REPLICATION: PRONIA**									
**VARIABLES**	**ANOVA**			**PRONIA-NC vs PRONIA-PSY**		**PRONIA -NC vs PRONIA -STPS**		**PRONIA -PSY vs PRONIA -STPS**	
	**F (2, 368)**	**P**	**pFDR**	**t(280)**	**P**	**t(289)**	**P**	**t(167)**	**P**
DC META-ROI 2 (RH)	5.98	0.01	0.04	3.61	0.01	1.20	0.23	-1.78	0.07
DSST-ICM	58.23	0.01	0.01	-10.48	0.01	-6.19	0.01	3.70	0.01
ADS-CT	14.91	0.01	0.01	5.70	0.01	1.43	0.48	-3.22	0.01
**EXTERNAL REPLICATION: LIBD**									
**VARIABLES**	**ANOVA**			**LIBD-NC vs LIBD -PSY**		**LIBD -NC vs LIBD -SIB**		**LIBD -PSY vs LIBD -SIB**	
	**F (2, 229)**	**P**	**pFDR**	**t(201)**	**P**	**t(176)**	**P**	**t(81)**	**P**
WCST-TE	34.90	0.01	0.01	-7.46	0.01	1.86	0.06	4.29	0.01
WMS-TS	F(2, 139) = 31.07	0.01	0.01	t(120) = 7.35	0.01	t(96) = - 0.16	0.99	t(62) = - 4.45	0.01

Abbreviations: MI-NCR: Neurotypical controls in within-site replication cohort of the main investigation study; MI-PSY: Chronic patients with psychosis of the main investigation study; MI-STPS: Individuals with subthreshold psychotic symptoms of the main investigation study; PRONIA-NC: Neurotypical controls in PRONIA dataset; PRONIA-PSY: Patients with psychosis in PRONIA dataset; PRONIA-STPS: Individuals with subthreshold psychotic symptoms in PRONIA dataset; LIBD-NC: Neurotypical controls in LIBD dataset; LIBD-PSY: Patients with psychosis in LIBD dataset; LIBD-SIB: Unaffected siblings of patients with schizophrenia in LIBD dataset; BC: Betweenness centrality; DC: Degree centrality; LH: Left hemisphere; RH: right hemisphere; WCST-TE: Number of total errors at Wisconsin Card Sorting Test; NB-EI: N-Back efficiency index; WMS-TS: Wechsler Memory Scale - Total score; TMT-BA: Trail Making Test - difference between Part B and part A; CPT-FA: Continuous Performance Test - False alarms ratio; DSST-ICM: Inverse number of correct symbol-number correspondences at Digit Symbol Substitution Test; ADS-CT: Number of correct trials at Auditory Digit Span.

Table S4: Comparison between cohorts consisted of individuals with subthreshold psychotic symptoms.

SYMPTOMS	MI-STPS	PNC-STPS	PRONIA-STPS	MI-STPS/ PNC-STPS	MI-STPS/ PRONIA-STPS	PNC-STPS / PRONIA-STPS
**Psychotic symptoms**	6	12	1	NS	$\chi 2$ = 16.2**	$\chi 2$ = 19.4**
**Anxiety symptoms**	6	10	14	NS	NS	NS
**Manic symptoms**	3	13	2	NS	NS	$\chi 2$ = 18.7**
**Depressive symptoms**	3	10	44	NS	$\chi 2$ = 7.8*	$\chi 2$ = 9.2*

*p<0.005; **p<0.001

In the left box, the table shows the psychopathological domain prevalent in each cohort consisted of individuals with subthreshold psychotic symptoms. In the left panel, Chi Squared statistics of the comparison between cohorts consisted of individuals with subthreshold psychotic symptoms are listed. Abbreviation: MI-STPS: Individuals with subthreshold psychotic symptoms of main investigation; PNC-STPS: Individuals with a developmental trajectory toward psychotic disorders of Philadelphia Neurodevelopmental Cohort; PRONIA-STPS: Individuals with subthreshold psychotic symptoms of PRONIA study: NS: not significant.

Table S5: Sex-related differences in the brain-behaviour association.

Cohort	DC & executive performance in MALE					DC & executive performance in FEMALE				
	**N**	**r**	**P**	**DoF**	**Cov**	**N**	**r**	**P**	**DoF**	**Cov**
**MI-NCD**	57	0.37	0.01	54	Age	60	0.29	0.01	57	Age
**MI-NCR**	42	0.26	0.05	38	Age and TR	45	0.27	0.04	41	Age and TR
**MI-PSY**	32	-0.34	0.03	28		11	-0.30	0.22	7	
**MI-STPS**	9	-0.26	0.29	5		13	-0.71	0.01	9	
**PNC-TD**	34	0.16	0.19	31	Age	23	0.49	0.01	20	Age
**PNC-STPS**	17	-0.38	0.07	14		28	-0.18	0.19	25	
**PNC-OD**	95	0.18	0.09	92		134	0.01	0.99	131	
**PRONIA-NC**	72	0.15	0.10	67	Age and MRI scanner	130	0.11	0.12	126	Age and MRI scanner
**PRONIA-PSY**	36	-0.08	0.33	31		44	-0.41	0.01	40	
**PRONIA-STPS**	43	-0.05	0.37	38		46	0.22	0.08	42	
**LIBD-NC**	94	0.09	0.20	91	Age	55	0.31	0.01	52	Age
**LIBD-PSY**	34	-0.13	0.23	31		20	-0.58	0.01	17	
**LIBD-SIB**	15	0.08	0.79	12		14	0.03	0.92	11	

Results of Pearson's partial correlation analyses, investigating potential sex-related differences in the association between the degree centrality of the prefrontal-cingulate-striatal meta-ROI and the executive performance across each study cohort. Abbreviations: MI-NCD: Neurotypical controls in the discovery cohort of the main investigation; MI-NCR: Neurotypical controls in within-site replication cohort of the main investigation; MI-PSY: Chronic patients with psychosis of the main investigation; MI-STPS: Individuals with subthreshold psychotic symptoms of the main investigation; PNC-TD: Individuals with typical development in PNC dataset; PNC-STPS: Individuals with a developmental trajectory towards psychotic disorders in PNC dataset; PNC-OD: Individuals with a developmental trajectory towards other psychiatric disorders in PNC dataset; PRONIA-NC: Neurotypical controls in PRONIA dataset; PRONIA-PSY: Patients with a recent-onset psychosis in PRONIA dataset; PRONIA-STPS: Individuals with subthreshold psychotic symptoms in PRONIA dataset; LIBD-NC: Neurotypical controls in LIBD dataset; LIBD-PSY: Patients with schizophrenia in LIBD dataset; LIBD-SIB: Unaffected siblings of patients with schizophrenia in LIBD dataset; N: Number of subjects included in the cohort; DoF: Degree of freedom; Cov: Covariates included in the Pearson's partial correlation analyses; TR: Repetition time.

References

[1] M. First, M. Gibbon, R. L. Spitzer, J. J. N. Y. B. R. D. Williams, New York State Psychiatric Institute, **1996**.

[2] F. Schultze-Lutter, J. Addington, S. Ruhrmann, J. J. R. G. F. Klosterkötter, **2007**.

[3] T. McGlashan, B. Walsh, S. Woods, *The psychosis-risk syndrome: handbook for diagnosis and follow-up*, Oxford University Press, **2010**.

[4] L. A. Antonucci, A. Raio, G. Pergola, B. Gelao, M. Papalino, A. Rampino, I. Andriola, G. Blasi, A. J. B. p. Bertolino, **2021**, *9* (1), 1.

[5] R. C. Gur, M. E. Calkins, T. D. Satterthwaite, K. Ruparel, W. B. Bilker, T. M. Moore, A. P. Savitt, H. Hakonarson, R. E. J. J. p. Gur, **2014**, *71* (4), 366.

[6] R. M. Xavier, M. E. Calkins, D. S. Bassett, T. M. Moore, W. T. George, J. H. Taylor, R. E. Gur, *Issues in mental health nursing* **2022**, 1, https://doi.org/10.1080/01612840.2022.2099494.

[7] N. Koutsouleris, L. Kambeitz-Ilankovic, S. Ruhrmann, M. Rosen, A. Ruef, D. B. Dwyer, M. Paolini, K. Chisholm, J. Kambeitz, T. J. J. p. Haidl, **2018**, *75* (11), 1156.

[8] J. J. Randolph, K. J. P. A. Falbe, Research, Evaluation, **2014**, *19*.

[9] R. K. J. P. a. r. Heaton, **1981**.

[10] F. Yang, H. Ma, J. Yuan, Y. Wei, L. Xu, Y. Zhang, C. Kang, J. J. P. r. Yang, **2021**, *299*, 113862.

[11] a) J. H. Callicott, V. S. Mattay, A. Bertolino, K. Finn, R. Coppola, J. A. Frank, T. E. Goldberg, D. R. J. C. c. Weinberger, **1999**, *9* (1), 20; b) A. S. Gevins, S. L. Bressler, B. A. Cutillo, J. Illes, J. C. Miller, J. Stern, H. R. J. E. Jex, c. neurophysiology, **1990**, *76* (4), 339.

[12] G. Pergola, P. Di Carlo, E. D'Ambrosio, B. Gelao, L. Fazio, M. Papalino, A. Monda, G. Scozia, B. Pietrangelo, M. J. T. p. Attrotto, **2017**, *7* (1), e1006.

[13] D. J. T. J. o. P. Wechsler, **1945**, *19* (1), 87.

[14] L. A. Antonucci, G. Pergola, A. Pigoni, D. Dwyer, L. Kambeitz-Ilankovic, N. Penzel, R. Romano, B. Gelao, S. Torretta, A. J. B. p. Rampino, **2020**, *87* (8), 697.

[15] R. Reitan, D. Wolfson, The halstead-reitan cognitive test battery: Theory and clinical interpretation. Neuropsychology Press, Tucson, AZ: **1993**.

[16] J. Lee, S. J. S. R. Park, **2006**, *81* (2-3), 191.

[17] M. M. Kurtz, B. E. Wexler, M. D. J. S. R. Bell, **2004**, *68* (1), 95.

[18] J. J. J. o. c. p. Jaeger, **2018**, *38* (5), 513.

[19] J. Ashburner, K. J. J. N. Friston, **2005**, *26* (3), 839.

[20] a) L. L. Backhausen, M. M. Herting, J. Buse, V. Roessner, M. N. Smolka, N. C. J. F. i. n. Vetter, **2016**, *10*, 558; b) M. Reuter, M. D. Tisdall, A. Qureshi, R. L. Buckner, A. J. van der Kouwe, B. J. N. Fischl, **2015**, *107*, 107.

[21] Z. Song, N. Tustison, B. Avants, J. C. Gee, in *International conference on medical image computing and computer-assisted intervention* Springer, **2006**, 831-838.

[22] a) L. A. Antonucci, L. Fazio, G. Pergola, G. Blasi, G. Stolfa, P. Di Palo, A. Mucci, P. Rocca, C. Brasso, M. J. S. r. di Giannantonio, **2022**, *240*, 193; b) R. Passiatore, L. A. Antonucci, T. P. DeRamus, L. Fazio, G. Stolfa, L. Sportelli, G. C. Kikidis, G. Blasi, Q. Chen, J. J. P. o. t. N. A. o. S. Dukart, **2023**, *120* (32), e2221533120; c) T. D. Satterthwaite, M. A. Elliott, K. Ruparel, J. Loughead, K. Prabhakaran, M. E. Calkins, R. Hopson, C. Jackson, J. Keefe, M. J. N. Riley, **2014**, *86*, 544.

[23] J. M. Schwarzer, I. Meyhoefer, L. A. Antonucci, L. Kambeitz-Ilankovic, M. Surmann, O. Bienek, G. Romer, U. Dannlowski, T. Hahn, A. J. N. Korda, **2022**, *47* (12), 2051.

[24] T. D. Satterthwaite, S. N. Vandekar, D. H. Wolf, D. S. Bassett, K. Ruparel, Z. Shehzad, R. C. Craddock, R. T. Shinohara, T. M. Moore, E. D. J. M. p. Gennatas, **2015**, *20* (12), 1508.

[25] K. J. Friston, S. Williams, R. Howard, R. S. Frackowiak, R. J. M. r. i. m. Turner, **1996**, *35* (3), 346.

[26] A. X. Patel, P. Kundu, M. Rubinov, P. S. Jones, P. E. Vértes, K. D. Ersche, J. Suckling, E. T. J. N. Bullmore, **2014**, *95*, 287.

[27] J. D. Power, K. A. Barnes, A. Z. Snyder, B. L. Schlaggar, S. E. J. N. Petersen, **2012**, *59* (3), 2142.

[28] N. U. Dosenbach, B. Nardos, A. L. Cohen, D. A. Fair, J. D. Power, J. A. Church, S. M. Nelson, G. S. Wig, A. C. Vogel, C. N. J. S. Lessov-Schlaggar, **2010**, *329* (5997), 1358.

[29] Z. Yao, B. Hu, Y. Xie, P. Moore, J. J. B. i. Zheng, **2015**, *2* (1), 45.

[30] a) S. Guo, N. He, Z. Liu, Z. Linli, H. Tao, L. J. T. C. J. o. P. Palaniyappan, **2020**, *65* (1), 21; b) S. S. Haas, L. A. Antonucci, J. Wenzel, A. Ruef, B. Biagianti, M. Paolini, B.-S. Rauchmann, J. Weiske, J. Kambeitz, S. J. N. Borgwardt, **2021**, *46* (4), 828; c) P. Jain, A. K. Sao, A. S. Minhas, in *2021 43rd Annual International Conference of the IEEE Engineering in Medicine & Biology Society (EMBC)* IEEE, **2021**, 6695-6698; d) R. Prajapati, I. A. J. I. J. o. N. Emerson, **2021**, *131* (2), 105.

[31] M. Gajdoš, E. Výtvarová, J. Fousek, M. Lamoš, M. J. B. t. Mikl, **2018**, *31* (5), 767.

[32] J. D. J. N. C. Medaglia, **2017**, *27* (4), 593.

[33] a) J. D. Power, B. L. Schlaggar, C. N. Lessov-Schlaggar, S. E. J. N. Petersen, **2013**, *79* (4), 798; b) O. J. D. i. C. N. Sporns, **2013**, *15* (3), 247.

[34] A. Salavaty, M. Ramialison, P. D. Currie, *Patterns (New York, N.Y.)* **2020**, *1* (5), 100052, https://doi.org/10.1016/j.patter.2020.100052.

[35] J. Kruschwitz, D. List, L. Waller, M. Rubinov, H. J. J. o. n. m. Walter, **2015**, *245*, 107.

[36] a) E. Rikandi, T. Mäntylä, M. Lindgren, T. Kieseppä, J. Suvisaari, T. T. Raij, *Schizophrenia research* **2022**, *241*, 83, https://doi.org/10.1016/j.schres.2022.01.006; b) M. Rubinov, O. Sporns, *NeuroImage* **2010**, *52* (3), 1059, https://doi.org/10.1016/j.neuroimage.2009.10.003.

[37] Y. Du, Z. Fu, J. Sui, S. Gao, Y. Xing, D. Lin, M. Salman, A. Abrol, M. A. Rahaman, J. J. N. C. Chen, **2020**, *28*, 102375.

[38] a) N. Tzourio-Mazoyer, B. Landeau, D. Papathanassiou, F. Crivello, O. Etard, N. Delcroix, B. Mazoyer, M. Joliot, *NeuroImage* **2002**, *15* (1), 273, https://doi.org/10.1006/nimg.2001.0978; b) C. A. Pedersini, J. Guàrdia-Olmos, M. Montalà-Flaquer, N. Cardobi, J. Sanchez-Lopez, G. Parisi, S. Savazzi, C. A. Marzi, *PLoS One* **2020**, *15* (1), e0226816, https://doi.org/10.1371/journal.pone.0226816.

[39] A. Škoch, B. Rehák Bučková, J. Mareš, J. Tintěra, P. Sanda, L. Jajcay, J. Horáček, F. Španiel, J. Hlinka, *Scientific data* **2022**, *9* (1), 486, https://doi.org/10.1038/s41597-022-01596-9.
